# Early postlesional visuomotor capacity after V1 damage in the macaque

**DOI:** 10.1093/cercor/bhag138

**Published:** 2026-10-05

**Authors:** Alessia Sepe, Matteo Panormita, Mark A G Eldridge, David A Leopold, Luca Bonini, Wim Vanduffel, Marco Tamietto

**Affiliations:** Department of Neurosciences, Laboratory of Neuro- and Psychophysiology, KU Leuven Medical School, Herestraat 49, Box 1027, 3000 Leuven, Belgium; Leuven Brain Institute, KU Leuven, Herestraat 49, Box 1020, 3000 Leuven, Belgium; Department of Medicine and Surgery, Neuroscience Unit, University of Parma, Via Volturno 39/E, Gramsci 14, 43126 Parma, Italy; Department of Neurosciences, Laboratory of Neuro- and Psychophysiology, KU Leuven Medical School, Herestraat 49, Box 1027, 3000 Leuven, Belgium; Leuven Brain Institute, KU Leuven, Herestraat 49, Box 1020, 3000 Leuven, Belgium; Department of Psychology and Neuroscience Institute of Turin (NIT), University of Torino, Via Giuseppe Verdi 10, 10124 Torino, Italy; Biosciences Institute, Newcastle University, Framlington Place, Newcastle upon Tyne, NE2 4HH, United Kingdom; Section on Cognitive Neurophysiology and Imaging, National Institute of Mental Health, 9000 Rockville Pike, Bethesda, MD 20892, United States; Section on Cognitive Neurophysiology and Imaging, National Institute of Mental Health, 9000 Rockville Pike, Bethesda, MD 20892, United States; Neurophysiology Imaging Facility, National Institute of Mental Health, National Institute of Neurological Disorders and Stroke, National Eye Institute, 9000 Rockville Pike, Bethesda, MD 20892, United States; Department of Medicine and Surgery, Neuroscience Unit, University of Parma, Via Volturno 39/E, Gramsci 14, 43126 Parma, Italy; Department of Neurosciences, Laboratory of Neuro- and Psychophysiology, KU Leuven Medical School, Herestraat 49, Box 1027, 3000 Leuven, Belgium; Leuven Brain Institute, KU Leuven, Herestraat 49, Box 1020, 3000 Leuven, Belgium; Athinoula A. Martinos Center for Biomedical Imaging, Massachusetts General Hospital, 149 13th Street, Charlestown, MA 02129, United States; Department of Radiology, Harvard Medical School, Boston, 25 Shattuck Street, Boston, MA 02144, United States; Department of Psychology and Neuroscience Institute of Turin (NIT), University of Torino, Via Giuseppe Verdi 10, 10124 Torino, Italy; Department of Medical and Clinical Psychology, Tilburg University, Warandelaan 2, P.O. Box 90153, 5000 LE, 5037 AB Tilburg, The Netherlands; Italian Academy for Advanced Studies in America, Columbia University, New York, 1161 Amsterdam Avenue, New York, NY 10027, United States

**Keywords:** blindsight, nonhuman primates, saccades, V1 lesion, visual perimetry

## Abstract

Damage to the primary visual cortex (V1) causes field defects in conscious vision, yet stimuli in the affected field may still guide saccades and other visuomotor responses (“blindsight”). Chronic blindsight is well documented, but early postlesional saccade profiles to scotoma stimuli remain poorly characterized. We mapped visuomotor functions in two macaques 3 to 4 weeks after a unilateral V1 lesion using a perimetry-like saccade assay up to 35° eccentricity, with analyses of saccade direction and catch-trial baselines. Targets had either unpredictable onset, as in clinical perimetry, or temporally cued onset. With unpredictable onsets, monkeys typically failed to initiate movements toward the affected hemifield, and correct saccades to scotoma targets were rare. Temporal cueing increased saccade initiation toward contralesional targets, including positions meeting conventional criteria for blindness, primarily as delayed saccades with initial misdirection or multistep trajectories. A scotoma-related spatial bias, largely independent of the target, was evident in catch-trial and misdirected saccades, whereas a minority of delayed (“out-of-time”) saccades reached toward the target location without meeting the temporal criterion. Fine-grained saccade phenotyping may offer a sensitive marker of residual visuomotor processing after V1 damage in monkeys and, potentially, humans.


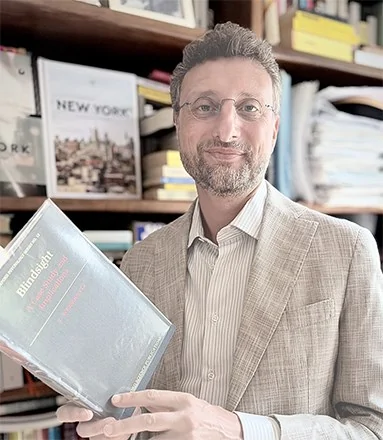


**Marco Tamietto** is a Professor of Cognitive and Affective Neuroscience at the University of Turin, Italy, where he directs the Neuroscience Institute, and a Research Fellow at Tilburg University, the Netherlands. He has held visiting fellowships at the Royal Netherlands Academy of Arts and Sciences (2018), the Accademia Nazionale dei Lincei (2021–2024), and the Italian Academy for Advanced Studies in America at Columbia University, New York (2026). His research investigates the organization of the visual system, the neural bases of consciousness and affective processing in humans and nonhuman primates. His work has received support from national and international fundings, including the European Research Council. He was awarded the Gold Medal of the Istituto Lombardo, Accademia di Scienze e Lettere, and is an Honorary Member of the Department of Experimental Psychology and a member of the Senior Common Room at Worcester College, University of Oxford.

## Introduction

Visual events can influence behavior even in the absence of subjective visual experience. Damage to the primary visual cortex (V1) causes clinical blindness in the retinotopic locations served by the damaged tissue. However, patients can sometimes detect, localize, or discriminate stimuli they report not seeing when forced-choice procedures are used (Pöppel et al. 1973; Weiskrantz et al. 1974; Zihl and von Cramon, 1980). This dissociation between the absence of visual awareness and residual visuomotor performance is referred to as “blindsight” (Weiskrantz et al. 1974; Azzopardi and Cowey 1998; Van den Stock et al. 2015;  Danckert et al. 2019)*.* Analogous dissociations have been documented in nonhuman primates; macaques with V1 lesions can still orient, reach, or move their eyes toward stimuli presented in the affected visual field (Mohler and Wurtz 1977; Cowey and Stoerig 1995; Moore et al. 1995; Striemer, Chapman and Goodale 2009;  Schmid et al. 2010; Isa and Yoshida 2021). Because primate visual systems are evolutionarily conserved and share homologous cortico-subcortical pathways, macaque models provide a valuable framework for studying how residual visual signals can guide action when the primary geniculo-striate pathway is compromised (Armendariz et al. 2019; Celeghin et al. 2019; Kletenik et al. 2022; Yu et al. 2024). Moreover, a “precision” animal model of blindsight can also help disentangle different accounts of postlesion compensation and recovery (Tamietto and Morrone 2016; Fox et al. 2020).

A central challenge when studying blindsight in nonhuman primates is that conscious vision cannot be assessed through verbal reporting, and subjects cannot explicitly deny perceiving stimuli to which they respond (Overgaard 2011; Sahraie et al. 2013). Nevertheless, blindsight in macaques has been operationally defined as the dissociation between visual judgments made under two paradigms: an “uncued” detection task and a temporally “cued” detection task (Moore et al. 1995; Yoshida and Isa 2015). In the uncued yes/no paradigm (hereafter, “fixation-ON task”), targets occur unpredictably across different locations in the visual field while the central fixation remains present - a design analogous to clinical perimetry in humans. Failure to orient is operationally interpreted as evidence of cortical blindness and is used to delineate the scotoma. In the temporally cued paradigm (hereafter “fixation-OFF task”), the target onset is signaled by the fixation offset, encouraging a “guess-like” response even when the target is not consciously perceived. The dissociation between the two paradigms can be remarkable: with identical stimuli, the same subject may perform at chance level (~50%) in an uncued yes/no context, yet exceed 90% accuracy under forced-choice conditions that compel a response, or show increased responding when a temporal cue reduces uncertainty about target onset (Cowey and Stoerig 1995; Azzopardi and Cowey 1997, 1998; Isa and Yoshida 2021).

The behavioral phenomenology of blindsight and its relation to postlesion circuit dynamics remain incompletely understood. Three issues are especially relevant for interpreting visuomotor behavior after V1 damage and can be productively addressed in macaques by exploiting the dissociation between uncued yes/no and cued response paradigms. First, the temporal course of residual visuomotor functions is poorly characterized in the early postlesion period. In macaques, recovery of stimulus-guided saccades toward the affected hemifield often requires several weeks (Mohler and Wurtz 1977; Yoshida et al. 2008), and behavioral testing begins only after performance has stabilized, typically months after the lesion (Cowey and Stoerig 1995; Schmid et al. 2010; Isa and Yoshida 2021). The residual capacities differ greatly depending on the developmental age of the lesion (Tinelli et al. 2013; Warner et al. 2015; Mundinano et al. 2018; Bourne et al. 2024).

Much less is known about the period immediately after a lesion is acquired in adulthood. One possibility is that V1-independent pathways rapidly compensate, supporting behavioral strategies used before the consolidation of longer-term plastic reorganization. Recent work in humans underscores the importance of this early window (Saionz et al. 2020). In patients studied during the subacute period after occipital stroke, perimetrically blind regions could still support measurable visual discriminations, including motion discrimination and contrast sensitivity, that were largely absent in chronic patients if left untrained. Moreover, training initiated subacutely was substantially faster, more effective, and more spatially generalizable than the same training initiated later. These observations make the early postlesion period especially informative for distinguishing between preexisting V1-independent contributions and postlesion circuit reorganization and remodeling as sources of residual behavior (Tamietto and Morrone 2016; Fox et al. 2020).

Second, the spatial ecology of residual visuomotor functions has often been assessed over a limited portion of the visual field, with an emphasis on parafoveal and mid-peripheral targets, potentially underestimating the importance of the visual periphery in natural orienting. Peripheral vision may be particularly important for rapid responses to sudden events, looming, self-motion stabilization, and continuous updating of head/body orientation (Mikellidou et al. 2017). Recent evidence challenges the assumption that the principles underlying central and eccentric vision are equivalent. For example, eccentric vision recruits partially distinct computations and relies more strongly on subcortical contributions that remain available after V1 injury (e.g., via superior colliculus and thalamic relays), as well as on distinct cortical nodes in the dorsal stream (Mohler and Wurtz 1977; Hasson et al. 2002; Kaas and Lyon 2007; Fattori et al. 2009; Schmid et al. 2010; Kinoshita et al. 2019; Barbot et al. 2021). Therefore, a wide-field approach examining visual loss also in the far periphery may be essential to capture a fuller spatial profile of early residual orienting and different modes of natural behavior.

Third, visually guided saccades involve distinct components, including target detection and selection, evidence accumulation, motor planning and initiation, and trajectory control. Each of these processes could be variably impacted by V1 damage and may recover differently. Area V1 provides a prominent, topographically organized projection to the superior colliculus, which influences saccade generation and cortical oculomotor networks (Hafed et al. 2023). Standard scoring metrics that reduce oculomotor responses to “hit vs. miss” or endpoint deviation from target location may overlook subtle informative patterns embedded in non-normative saccades. Specifically, atypical eye movements, such as delayed saccades, initial misdirections followed by corrections, or multistep/curved trajectories, are generally treated as noise or classified as errors (Mohler and Wurtz 1977; Moore et al. 1995). A more nuanced approach to “saccade phenotyping” may instead reveal non-uniform, graded, or spatially specific residual visuomotor transformations early after V1 lesion.

The present study addresses these issues by conducting dense, wide-field mapping of saccadic behavior in 2 macaques tested within 1 month (3 to 4 weeks) after a unilateral V1 lesion. We employed a perimetry-like “fixation-ON” task to define field defects under unpredictable target onsets, and a fixation-OFF task in which fixation offset served as a temporal cue to target appearance. Although this differs structurally from forced-choice procedures (in which the subject selects among simultaneously presented alternatives), both share the aim of encouraging a response when the target is not consciously perceived, and temporal cueing has historically been used alongside forced-choice procedures to reveal blindsight in humans (Pöppel et al. 1973; Weiskrantz et al. 1974) and monkeys (Mohler and Wurtz 1977; Cowey and Stoerig 1995; Moore et al. 1995; Schmid et al. 2010; Isa and Yoshida 2021). Targets were sampled across eccentricities up to 35°, covering parafoveal, mid-peripheral, and far-peripheral locations. Crucially, we quantified both canonical, direct saccades and multiple classes of atypical oculomotor responses, including direct but late saccades that failed to meet reward criteria, saccades with initial trajectory deviations from the target, and compound, multistep, or curved trajectories, and saccades during target-absent catch trials.

Consistent with classic blindsight paradigms in chronic V1-damaged macaques (Moore et al. 1995), temporal cueing in the “fixation-OFF” task increased saccade initiation toward contralesional locations, primarily by converting “misses” into atypical responses (delayed or initially misdirected saccades). Critically, the ON–OFF dissociation was most apparent when we quantified atypical saccades (e.g., long-latency, misdirected, and multistep/curved trajectories), revealing spatially structured residual visuomotor influences that would be overlooked by conventional correct/miss scoring. Together, these findings indicate that within weeks of V1 injury, residual V1-independent visuomotor signals and oculomotor biases toward scotoma boundaries can still shape saccade initiation and coarse orienting across the scotoma, even though precise and timely guidance remains impaired and qualitatively different from chronic blindsight.

## Materials and methods

### Subjects

Two adult male rhesus macaques (*Macaca mulatta*; 7 to 10 kg; referred to here as M1 and M2) participated in this study. The animals were implanted with a custom polyetherimide headpost, secured with ceramic screws and dental acrylic to stabilize head position during training and recording (Vanduffel et al. 2001).

All procedures complied with the EU Directive 2010/63/EU and were approved by the KU Leuven Animal Ethics Committee (protocol number P173/2020). Animal housing and handling followed the recommendations of the Weatherall report. Monkeys were group-housed in enriched cages with a minimum volume of 16 to 32 m^3^, promoting social interactions and locomotor behavior, at the KU Leuven Medical School primate facility. Environmental enrichment items, such as wood, ropes, toys, and foraging devices, were provided, and the animals were kept on a 12-h light/dark cycle with either natural or monitored artificial light. Animals received standard primate chow, supplemented daily with fruit and other foods such as vegetables, bread, and dried fruit. Food was available ad libitum; water and fluid access followed facility and experimental protocols, with task-related fluid earned as a reward during experimental sessions based on performance.

Data from the two animals involved in this experiment have been reported previously under different identifiers (M60 and M61; here M1 and M2, respectively).

### Unilateral V1 lesion

A unilateral, partial aspiration lesion of the right V1 was performed under aseptic conditions, guided by pre-operative T1-weighted structural magnetic resonance imaging (MRI; 3D MPRAGE sequence; TR = 2,700 ms; TE = 3.41 ms; in-plane FOV = 104 × 128 mm; matrix = 260 × 320; slices = 208; flip angle α = 9°; TI = 882 ms) (Fig. 1A). Anesthesia was induced with ketamine (Nimatek, 100 mg/ml; 10 mg/kg^−1^ i.m.) and medetomidine (Domitor, 1 mg/ml; 4.2 mg/kg^−1^ i.m.) and maintained with propofol (Propovet, 10 mg/ml; 10 to 12.5 mg/kg/hour i.v.). Vital parameters (heart rate, SpO₂, end-tidal CO₂, and body temperature) were continuously monitored throughout the procedure.

**Figure 1:**
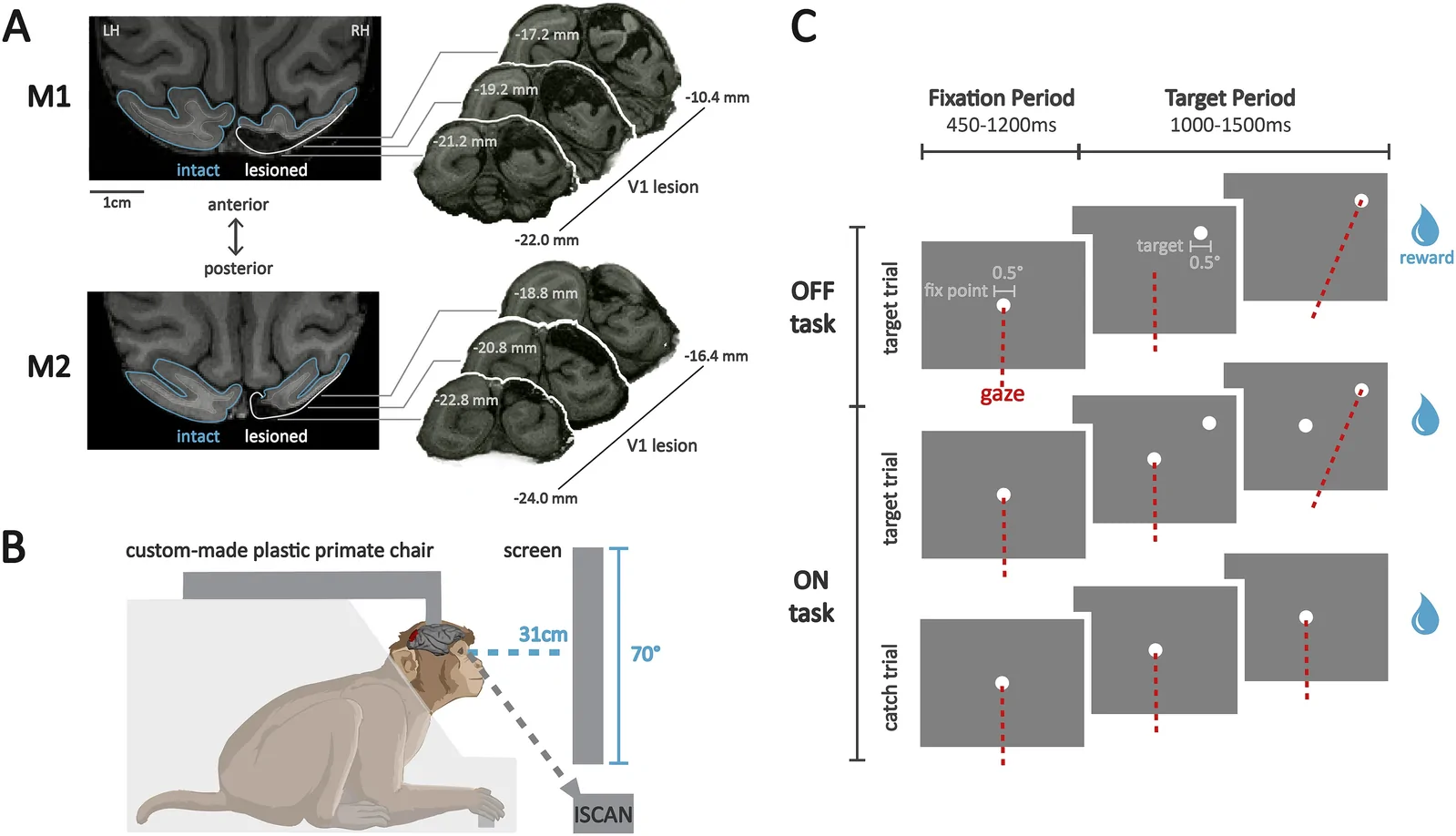
Experimental design and task structure for saccade mapping after unilateral V1 lesion. A) Axial and coronal T1-weighted MRI slices showing right-hemisphere V1 aspiration lesion in M1 (top) and M2 (bottom). The blue contour indicates the prelesion boundary of V1 gray matter, and the white contour indicates the postlesion boundary on the lesioned hemisphere. B) Schematic of the experimental setup. Dotted lines indicate eye-to-screen and eye-to-camera (ISCAN eye-tracker) distances, allowing mapping of a ~70°-wide visual field. C) Trial sequence for the fixation-OFF (top) and fixation-ON (bottom) tasks. After a variable fixation period (450 to 1,200 ms), a 0.5° white dot served as a peripheral target during the target period (1,000 to 1,500 ms). In the fixation-OFF task, target onset was temporally cued by the simultaneous offset of the fixation dot (all trials were target trials). In the fixation-ON task, the fixation point remained visible throughout the trial, and target onset was unpredictable; catch trials were interleaved (20%). Red dashed traces illustrate the expected gaze movements; the blue drop indicates reward delivery contingent on meeting the task criteria for the respective trial type.

Following a ~4 cm occipital craniotomy, the dura was incised, and the cortex was aspirated along the dorso-caudal parts of the operculum and the dorsal bank of the calcarine fissure, while sparing the foveal representation (~0–2° eccentricity), as confirmed by post-surgical MRI (Fig. 1A). After the lesion, the dura was sutured, the bone flap was replaced and secured, and the overlying skin was closed.

Perioperative analgesia and care included meloxicam (Metacam, 5 mg/ml; 0.2 mg/kg^−1^ i.m., for 3 days), buprenorphine (Vetergesic, 0.3 mg/ml; 0.01 mg/kg^−1^ i.m., for 3 days), dexamethasone (Rapidexon, 2 mg/ml; 0.5 mg/kg^−1^ i.m., for 7 days), and cefazolin (Kefzol, 1 g; 50 mg/kg^−1^ i.m., for 15 days). Daily monitoring confirmed contra-lesional visual deficits alongside excellent overall health. Additionally, qualitative behavioral assessments in the home cage immediately after surgery (e.g., presentation of small objects and gradual hand movements entering the lower left visual quadrant from the periphery) were consistent with contralesional visual field loss.

### Experimental setup

Experiments took place in a dark, sound-attenuated room. Monkeys were trained using operant conditioning and positive reinforcement to perform visually guided saccade tasks while head-fixed in a natural “sphinx” posture in a custom-made primate chair (Vanduffel et al. 2001; Fig. 1B). Visual stimuli were displayed on a 54-inch LCD monitor (1,920 × 1,080; 60 Hz; Dell) placed 31 cm from the eyes, enabling stimulation and response mapping across a broad field of view up to 70° in diameter.

Eye position was continuously recorded from the right eye using an infrared corneal reflection system (Iscan, MA, USA; sampling rate 120 Hz) (Fig. 1B). The eye-tracking camera was mounted directly below the screen and centered on the tracked eye. Stimulus presentation, behavioral event detection, and reward delivery were controlled and synchronized with custom hardware and software to ensure precise temporal alignment and coregistration of all trial events. The screen background was set to RGB (128, 128, 128), corresponding to approximately 47.7 cd/m^2^ on the calibrated display. Saccade targets were white circular stimuli, 0.5° in diameter, set to RGB (255, 255, 255), corresponding to 130 cd/m^2^. The fixation point was also set to RGB (255, 255, 255).

### Behavioral tasks

Behavioral testing was conducted both before and approximately 3 weeks after the unilateral V1 lesion (pre- and postlesion testing durations: M1, about 2 weeks; M2, around 1 week). Two visually guided saccade paradigms were used (Fig. 1C): (i) an uncued yes/no detection task with unpredictable target onset (fixation-ON task), and (ii) a temporally cued task in which target onset coincided with the disappearance of the central fixation spot (fixation-OFF task). Aside from the presence or absence of the temporal cue (and catch trials; see below), task structure and stimuli were identical across paradigms (Fig. 1C).

#### Stimuli and sampling of visual space

In both tasks, monkeys fixated on a central white dot (0.5° diameter) and performed saccades to a peripheral target identical in size and color. Target locations were sampled across 7 eccentricities (5° to 35°, in 5° steps) and 12 polar angles (30° steps). Due to constraints of the setup (eye-screen distance and eye-tracker placement), three upper-field positions could not be reliably tested (30° and 35° on the upper vertical meridian, and 35° at the polar angle 30° clockwise from the upper vertical meridian), resulting in 81 target locations. Target positions were presented randomly with uniform sampling, so each location was tested the same number of times within a session (typically 5 times for M1 and 25 times for M2). Fixation-ON and fixation-OFF sessions were alternated on the same day to minimize confounds arising from time and motivational state. Across animals, the dataset included at least 40 (M1) and 30 trials per location (M2) in each task, both pre- and postlesion.

#### Temporal cueing and catch trials

In the fixation-OFF task (Moore et al. 1995; Fig. 1C, top), the fixation dot disappeared when the peripheral target appeared, providing an explicit temporal cue for saccade initiation; all trials included a target (i.e., no catch trials). In the fixation-ON task (Yoshida et al. 2008; Yoshida and Isa 2015; Takakuwa et al. 2023; Fig. 1C, bottom), the fixation dot remained visible throughout the trial, making target onset temporally unpredictable. Target trials were interleaved with catch trials (no target presented; 20% of trials).

#### Trial structure and performance criteria

Each trial began after a 500 ms inter-trial interval, with the onset of the central fixation dot. Monkeys were required to maintain fixation within a central virtual window (~3° × 3°) for a randomly variable interval (450 to 1,200 ms) to prevent timing-based anticipation (fixation period, Fig. 1C). Trials were aborted and discarded if fixation was broken during this period.

On target-present trials, following the fixation period, a peripheral target appeared and remained visible for a randomly variable duration of 1,000 to 1,500 ms (target period, Fig. 1C). Reward was delivered only if the monkey satisfied all three criteria within the same trial: (i) initiated an eye movement within the first 1,000 ms after target onset (response period), (ii) reached the target window within 200 ms after leaving the central fixation window, and (iii) then maintained gaze on the target window for at least 200 ms. Target windows were scaled with eccentricity (~3° × 3° for eccentricities 5° to 20°; ~4° × 4° for eccentricities 25° to 35°). Trials where any of these criteria were not met were unrewarded and classified according to the four behavioral categories defined in subsection Behavioral Outcome Categorization (Fig. 2): out-of-time responses (Fig. 2B), when the initial saccade was direct but failed to reach the target window within 200 ms; misdirected responses, when the initial saccade deviated from the target direction by ≥15° (Fig. 2C and D); and misses (Fig. 2E), when no eye movement was initiated within 1,000 ms after target onset.

**Figure 2:**
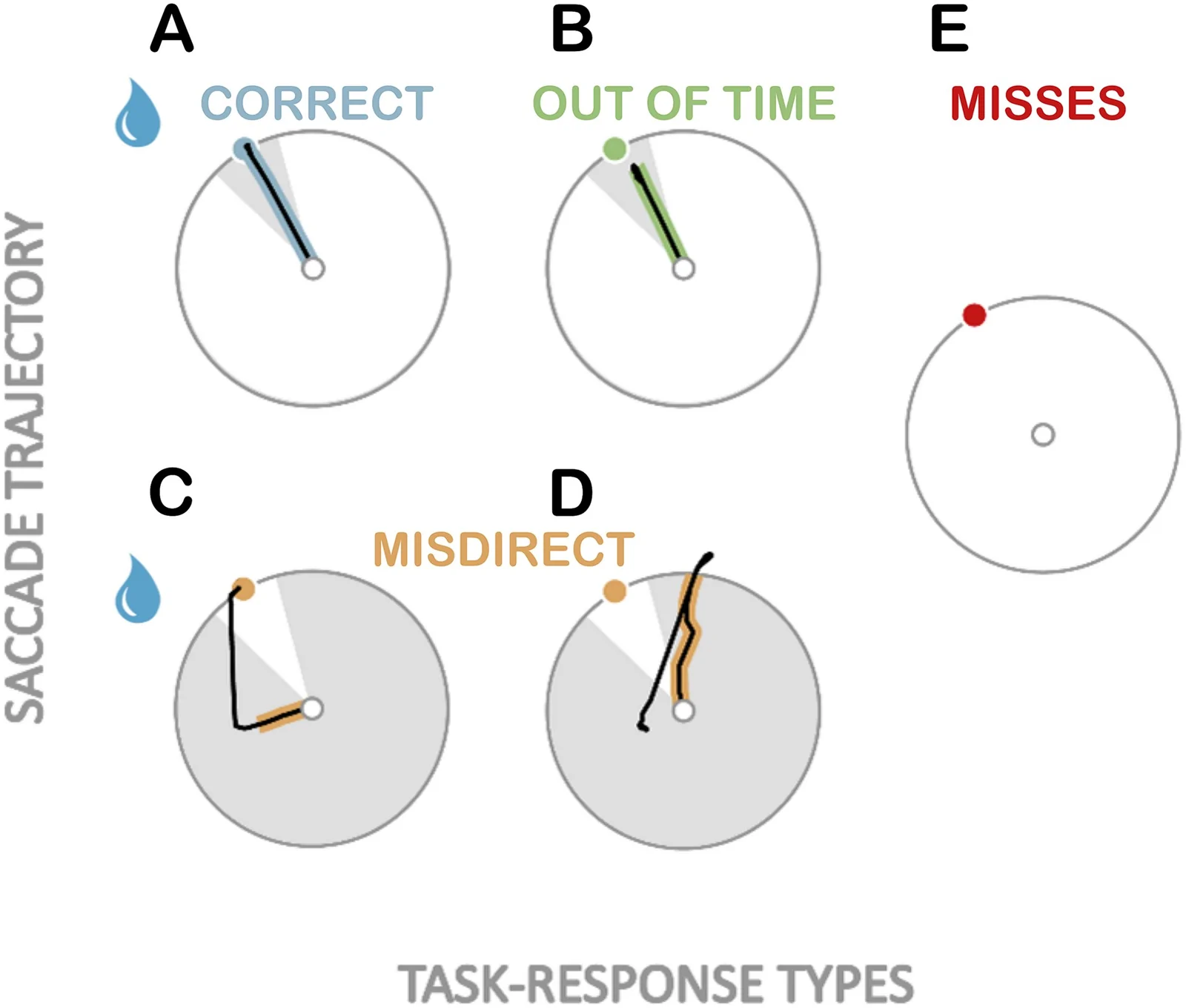
Behavioral outcome categories based on initial trajectory and response timing. Representative single-trial eye trajectories illustrating the four behavioral categories. Correct (A, blue), Out-of-time (B, green), Misdirect (C and D, yellow), and Misses (E, red). In each panel, the central open circle marks the fixation position, the colored dot marks the target location, the colored segment shows the first saccade (the segment used to compute angular deviation, AD), and the black trace shows the full gaze trajectory from fixation break to the end of the trial (including any corrective movement). Shaded sectors illustrate the ±15° AD criterion relative to the fixation-to-target vector; first saccades falling within this sector are classified as direct (AD < 15°; A and B), whereas those falling outside are classified as misdirected (AD ≥15°; C and D). Panels C and D show two common misdirect outcomes distinguished by task-defined response type: a misdirect trial that ultimately reaches the target and one that fails to reach the target within the response constraints. Misses (E) denote trials with no initiated eye movement within the response period. The water-drop symbol indicates a reward delivered when the trial meets the task’s criteria.

In fixation-ON catch trials, no peripheral target was presented after the fixation period. Fixation breaks during the corresponding response interval were treated as failed catch trials and subsequently used to estimate a target-independent saccade baseline. The labels “misdirect” and “miss” refer to task-defined reward outcomes; the final behavioral categories used for trajectory phenotyping additionally incorporated first-saccade direction and angular deviation, as described below.

Trials in which a misdirected first saccade was followed by corrective movements that ultimately entered the target window were not classified as correct in the subsequent trajectory-based analyses. Such trials were extremely rare (M1: 0.02% of scotoma-target trials; M2: 0.01%), and their contribution to the overall pattern of results is negligible. The 200 ms criterion, together with first-saccade-based classification, therefore serves a dual purpose: it rewards timely target acquisition and minimizes the possibility that performance reflects intact-field target detection following an initial saccade away from fixation.

### Data analysis

#### Eye-trace preprocessing and velocity-based analysis

Eye position signals (x and y coordinates) were converted from raw eye-tracker units to degrees of visual angle using a geometric calibration derived from task data. Specifically, for each target location, we computed the median gaze position when the eye was within the corresponding target acceptance window, and we fit a polynomial mapping from these gaze samples to the targets’ known screen coordinates. The resulting transformation was applied to all eye traces to express gaze in degrees of visual angle.

Instantaneous eye velocity was computed from the calibrated x-y traces, and velocity magnitude at each time $\left(v\ (t)\right)$ was defined as:


$$ v\ (t)=\sqrt{v_x\ {(t)}^2+{v}_y\ {(t)}^2\ } $$

where ${v}_x\ (t)$is the horizontal component of eye velocity at time *t* expressed in degrees/second (°/s), computed as the time derivative of horizontal position *x\ (t)*, and ${v}_y\ (t)$is the vertical component.

Based on velocity magnitude, each trial’s eye trace was segmented into three movement regimes: fixation (<20°/s), slow movement (20 to 100°/s), and saccade (>100°/s).

#### Definition of the initial saccade and endpoints

The onset of the initial saccade was defined as the moment when the eye first exited the central fixation window (i.e., fixation break). For trials in which the gaze eventually entered the target window, the saccade endpoint was defined as the last eye position within the target area, corresponding to the moment when velocity dropped below the saccade threshold (100°/s) near the target location.

Notably, the eye often executed multiple movements and did not reach the target in all trials. To ensure consistent classification across all trial outcomes, analyses of the initial trajectory geometry were restricted to the first saccadic segment, defined as the trajectory from the fixation break until the first time velocity dropped below 100°/s, regardless of where the eye landed.

#### Angular deviation analysis and trajectory classification

To determine whether the initial saccade was oriented toward the target, we measured how closely its direction matched the ideal target vector.

1. “Ideal direction''. For each target-present trial, we defined a reference vector from the saccade onset position (at the fixation break) to the target location, along the ideal straight-line path.
2. “Instantaneous direction''. Along the first saccadic segment, for each sampled eye position, we formed a vector from the saccade onset to that sample point.
3. “Angular Deviation (AD)''. At each sample point, we computed the angle between the instantaneous direction vector and the ideal target vector. The trial’s AD was the average of these angles across the first saccade segment.

Trials were classified as direct when AD < 15° and misdirected when AD ≥ 15°. The 15° criterion tolerates the mild curvature typical of normal visually guided saccades while identifying trials in which the initial movement direction was clearly inconsistent with the target direction.

The same threshold was applied uniformly across animals, tasks, eccentricities, and visual-field locations. Because catch trials contained no peripheral target and therefore no trial-specific target vector, they were not submitted to target-relative AD classification and were analyzed separately in absolute directional space.

#### Behavioral outcome categorization

Each target-present trial was classified into one of four behavioral categories, based on (i) the geometry of the first saccade (direct vs. misdirected) and (ii) whether the response achieved timely target acquisition within the task-defined temporal window.

1. “Correct” (Fig. 2A): The first saccade was direct (AD < 15°), and gaze entered the target window within 200 ms of the fixation break. This category includes both rewarded trials, in which target fixation was maintained for ≥200 ms, and trials in which the target was reached on time but fixation was not maintained long enough for reward.
2. “Out-of-time” (Fig. 2B): The first saccade was direct (AD < 15°) (i.e., directionally on target, but delayed or incomplete) but failed to enter the target window within 200 ms after the fixation break, indicating an initially target-directed response that did not satisfy the temporal criterion for timely target acquisition.
3. “Misdirect” (Fig. 2C and D): The first saccade was misdirected (AD ≥ 15°), often followed by additional movements. This category included both trials that ultimately reached the target window (C) and those that never did (D). Thus, trials were classified according to the first saccadic segment rather than the final endpoint of the full eye-movement sequence.
4. “Misses” (Fig. 2E): No saccade was initiated during the response period (1,000 ms after target onset), and the monkey maintained central fixation.

This categorization enabled us to map response profiles across the visual field and to quantify both canonical and atypical saccade phenotypes separately from the purely reward-based (Fig. 2A and C) and task-event labels (Fig. 2B and D).

#### Baseline target-independent saccade rate from catch trials

To estimate a lesion-related baseline of spontaneous (i.e., target-independent) saccades, we analyzed catch trials (no target) from postlesion fixation-ON sessions. For each monkey, we computed the proportion of catch trials in which fixation was broken (i.e., the eye left the central fixation window or a saccade was made outside it). This proportion was taken as an estimate of the baseline target-independent saccade rate (*p₀*) following the V1 lesion.

#### Test for target-evoked saccades above baseline in the fixation-ON task

We then tested whether, in the postlesion fixation-ON condition, targets at specific locations elicited saccades above *p₀*. For each target location (eccentricity × polar angle), target-present trials were grouped into those eliciting saccades (Correct + Out-of-time + Misdirect) versus those that did not (Miss). For each monkey and target location, we tested whether the probability of generating saccades when targets were presented (*p_target_*) exceeded the baseline target-independent saccade rate (*p₀*) using a one-sided exact binomial test (successes = target trials generating saccades; total = all target-present trials). The resulting *P*-values were corrected across target locations within each monkey using the Benjamini–Hochberg false discovery rate (FDR, α = 0.05).

#### *Bootstrap comparison of fixation-ON* versus *OFF tasks*

To quantify local, target-specific differences in response profiles between the fixation-OFF and fixation-ON tasks, we applied a nonparametric bootstrapping procedure separately for each monkey, target location, and response category (Correct, Out-of-time, Misdirect, Misses) (Python v3.12.2, incorporating SciPy v1.10.1, NumPy v1.24.3, and Pingouin v0.5.3). We adopted a nonparametric bootstrap because the response proportions were based on small, unequal trial counts per location, often included categories at or near zero (e.g., correct responses within the scotoma), and were compositional across the four mutually exclusive categories. Under these conditions, parametric tests that assume approximate normality or variance homogeneity are unreliable, whereas resampling from the empirical trial-level distribution requires no distributional assumptions and preserves the dependence among response categories. For each target location, we generated 1,000 bootstrap resamples by drawing trials with replacement within each task condition, while preserving the original number of trials per condition. For each bootstrap iteration, we computed the response proportion for each category and then calculated the paired difference (OFF–ON), yielding an empirical distribution of task effects.

Two-sided 95% confidence intervals were obtained from the 2.5th and 97.5th percentiles of the bootstrap difference distribution. Statistical significance was assessed using a two-sided bootstrap signed-rank test with


$$ p=2\times \mathit{\min}\ \{P (\varDelta \ge 0 ),P (\varDelta \le 0 \} $$

where *\varDelta* denotes the bootstrap OFF–ON difference. As a standardized effect size, we report


$$ t= mean\left(\varDelta \right)/ SE\left(\varDelta \right) $$

with $SE\left(\varDelta \right)$ defined as the standard deviation of the bootstrap difference distribution.

Bootstrap-derived *P*-values were FDR-corrected (Benjamini–Hochberg) across target locations. Analyses were first performed for locations within the perimetrically defined scotoma and then extended across the entire contralesional hemifield, regardless of fixation-ON performance, as a control and to generate full-field statistical maps (see Supplementary Material).

#### Directional analysis of atypical saccades and catch-trial saccades

To test whether atypical saccades carried directional information about target location, we analyzed three classes of postlesion fixation-ON saccades: Out-of-time responses, Misdirect responses, and saccades initiated on failed catch trials (hereafter, “catch saccades”). Catch trials, which contained no peripheral target, provided a benchmark for non–target-driven oculomotor behavior. The three classes were chosen because they span a continuum of expected target dependence: from Out-of-time (directionally on target, but delayed or incomplete) through Misdirect (initial trajectory inconsistent with the target) to catch saccades (no target on the screen).

##### Saccade-direction extraction

A uniform velocity-based pipeline was applied to all three response classes in absolute directional space. Two-dimensional eye velocity was computed from eye-position traces. Saccade onset was defined as the first sample following a fixation break at which eye velocity exceeded 100°/s. Saccade direction was computed as the angular displacement between eye position at onset and eye position 50 ms later, or at the end of the first velocity-defined segment if this occurred earlier. Angles were obtained from the four-quadrant arctangent and expressed as circular bearings In the range (0°, 360°), using the same polar-angle convention as the stimulus grid. Trials in which no post-fixation-break velocity-threshold crossing occurred, reflecting slow ocular drift rather than a ballistic saccade, were excluded from the directional analysis (M1: *n* = 10; M2: *n* = 10). This common pipeline was necessary because catch saccades lack a target and therefore cannot be analyzed using the target-relative angular-deviation procedure described in subsection Angular Deviation Analysis and Trajectory Classification.

##### Reference frames

Two reference frames were used. “Absolute direction” refers to saccade bearings in screen-centered polar coordinates and is defined for all three response classes. “Target-relative direction” was defined only for target-present trials (Out-of-time and Misdirect) as the signed circular difference between the saccade bearing and the target polar angle on the same trial, with 0° indicating a saccade aimed exactly at the target. Signed deviations were retained for circular statistics, so that the mean resultant vector represented the average directional offset from the target, while the mean resultant vector length ($ {\bar{R}} $) quantified directional concentration. For linear summaries (e.g., Mann–Whitney comparisons), absolute angular deviations were expressed in degrees, with 0° and 180° serving as reference points.

##### Global target-independent baseline (*p₀*)

The global false-alarm rate *p₀* used in the initiation-rate analysis of subsection Within-scotoma Saccade Initiation Exceeds the Aggregate Catch-trial Baseline was defined separately for each monkey, as the proportion of failed catch trials among all postlesion catch trials. Because catch trials contained no assigned target location, *p₀* is an aggregate target-independent baseline rather than a location-specific false-alarm map.

##### Tests of circular non-uniformity (within class)

Two complementary tests were used. The Rayleigh test (Rayleigh 1880; Fisher 1993) evaluates deviations from circular uniformity against a unimodal alternative. Rao’s spacing test (Rao 1976; Russell and Levitin 1995) detects non-uniformity without assuming unimodality and is therefore sensitive to bimodal or multimodal structure; a relevant feature given the broad and partly non-contiguous extent of the scotoma, especially in M1 (Landler et al. 2018). Both tests were applied to the absolute-space distributions of all three response classes. For the two target-present classes (Out-of-time and Misdirect), these tests were additionally applied in target-relative space.

##### Pairwise distribution comparisons (between classes)

Watson’s U^2^ test (Watson 1961, 1962), a rank-based nonparametric test that does not require uniformity or unimodality, was used to compare circular distributions. Within each monkey, U^2^ was computed for the three pairwise comparisons between absolute-space distributions of catch, Misdirect, and Out-of-time saccades, yielding six tests in total (three pairs × two monkeys). Critical values were taken from Zar (2010, Table B.43), *P*-values were obtained by interpolation, and values at or below the table minimum are reported as *P* ≤ 0.001. The six *P*-values were corrected for multiple comparisons using the Benjamini–Hochberg FDR procedure (Benjamini and Hochberg 1995).

##### Bias toward the scotoma border (binomial zone-proportion)

To assess whether saccades were overrepresented within predefined angular regions abutting the scotoma, we applied one-sided binomial tests against the geometric expectation derived from a uniform circular distribution. These tests were applied to Catch and Misdirect saccades in absolute directional space (i.e., the two classes used to characterize scotoma-border orienting that was independent of, or only weakly constrained by, the trial-specific target location in case of misdirected trials). Three nested angular scales were defined as symmetric margins around the polar angles occupied by scotoma target locations:

- “Target-angle zone” (±7.5°): concentration within the immediate vicinity of the sampled scotoma angles; the 7.5° half-width is the largest margin for which zones around adjacent target polar angles, spaced 30° apart, remain non-overlapping.
- “Narrow border zone” (±15°): one full grid step on either side, testing bias toward the scotoma boundary and its immediate surround.
- “Broad border zone” (±30°): two grid steps on either side, testing bias toward the wider scotoma-border region, including non-scotoma angles immediately adjacent to the defect.

For M1, zones were defined as the union of ±margin intervals around each of six scotoma anchor angles (90°, 120°, 150°, 180°, 210°, 240°) to accommodate the isolated affected angle at 90° in addition to the otherwise continuous 120° to 240° arc. For M2, zones were defined as a continuous arc spanning 180° to 240°, expanded symmetrically by the relevant margin, consistent with M2’s contiguous defect across this angular range. Null proportions were computed numerically at 1° resolution to account for interval overlap between adjacent anchor angles and for wrap-around at the 0°/360° boundary. All 12 binomial tests (2 monkeys × 2 response classes × 3 spatial scales) were corrected jointly with the Benjamini–Hochberg procedure.

##### Modal structure (von Mises mixture)

To characterize whether the absolute-space distributions of catch and Misdirect saccades were better described as unimodal or bimodal, we fitted von Mises mixture models with one or two components using maximum-likelihood estimation via the expectation–maximization algorithm. This analysis was restricted to Catch and Misdirect responses because its purpose was to describe target-independent or weakly target-constrained scotoma-border orienting in absolute visual-field coordinates. Model selection used the Bayesian Information Criterion (BIC), with the two-component model preferred when ΔBIC = BIC₁ − BIC₂ exceeded 2. For each selected model, we report the estimated modal directions, concentration parameters κ, and, for bimodal models, the mixing weight of the dominant component.

##### Robustness check using trajectory-based direction estimation

As a robustness check for target-present trials, the analyses in target-relative space were repeated using a direction estimator matched to the angular-deviation procedure of subsection Angular Deviation Analysis and Trajectory Classification. In this version, saccade onset was set to the fixation-break timestamp, and saccade direction was computed as the circular mean bearing of the instantaneous vectors from saccade onset to each sampled eye position across the full first saccadic segment (from fixation break until velocity returned below 100°/s). Target direction was referenced to the precise target location derived from its polar angle and eccentricity, matching the ideal target vector used for angular-deviation classification. Catch saccades were not included because no target was present.

##### Per-location regression not performed

We did not fit formal location-by-location regressions of Misdirect direction or amplitude against target polar angle or eccentricity. Although such models would directly address whether individual Misdirect responses scaled with target location, the observations were too sparse after stratification by monkey, task condition, response class, and target location to support stable per-location estimates.

## Results

### Perimetric mapping of the scotoma in the fixation-ON task

We defined the scotoma based on performance in the fixation-ON task, a perimetry-like condition in which the fixation point remained visible throughout the trial and target onset was therefore temporally uncued. To obtain a conservative estimate of visual loss, each target location was classified as scotoma only if postlesion performance showed the reciprocal pattern expected for blindness: an increase in misses relative to pre-lesion baseline together with a complementary reduction in correct responses. This criterion was applied only to miss and correct trials. Out-of-time and misdirected responses were not counted as misses because an eye movement was initiated, but they were also not counted as correct responses for the purpose of binary scotoma classification. They were therefore excluded from the miss/correct denominator used to define the scotoma operationally and analyzed separately as atypical saccadic outcomes in subsequent sections. As a result, locations dominated by out-of-time responses (e.g., 150°, 25 to 30° eccentricity in M1) could satisfy the scotoma criterion based on the miss/correct split alone, even when the majority of trials produced an atypical saccade. This procedure yielded an operational and spatially precise map of visual field impairment, analogous to clinical perimetry.

Both monkeys showed preserved saccadic localization in the ipsilesional (right) hemifield, with >90% correct responses and <10% misses, but showed marked impairments for contralesional (left) targets, often maintaining central fixation rather than initiating eye movements (Fig. 3A). In both animals, the operationally defined scotoma covered the lower-left quadrant across all tested eccentricities (5° to 35°). In M2, the scotoma formed a contiguous arc spanning polar angles 180° to 240°, sparing the upper vertical meridian, and contained an isolated preserved location at 10° eccentricity along the 180° meridian. In M1, the scotoma was more extensive, encompassing polar angles 90° to 240° across all tested eccentricities and additionally including an isolated affected region at the upper vertical meridian (120° polar angle), most prominently at 15° and 20° eccentricity. The spatial distribution of correct responses closely mirrored the scotoma.

**Figure 3:**
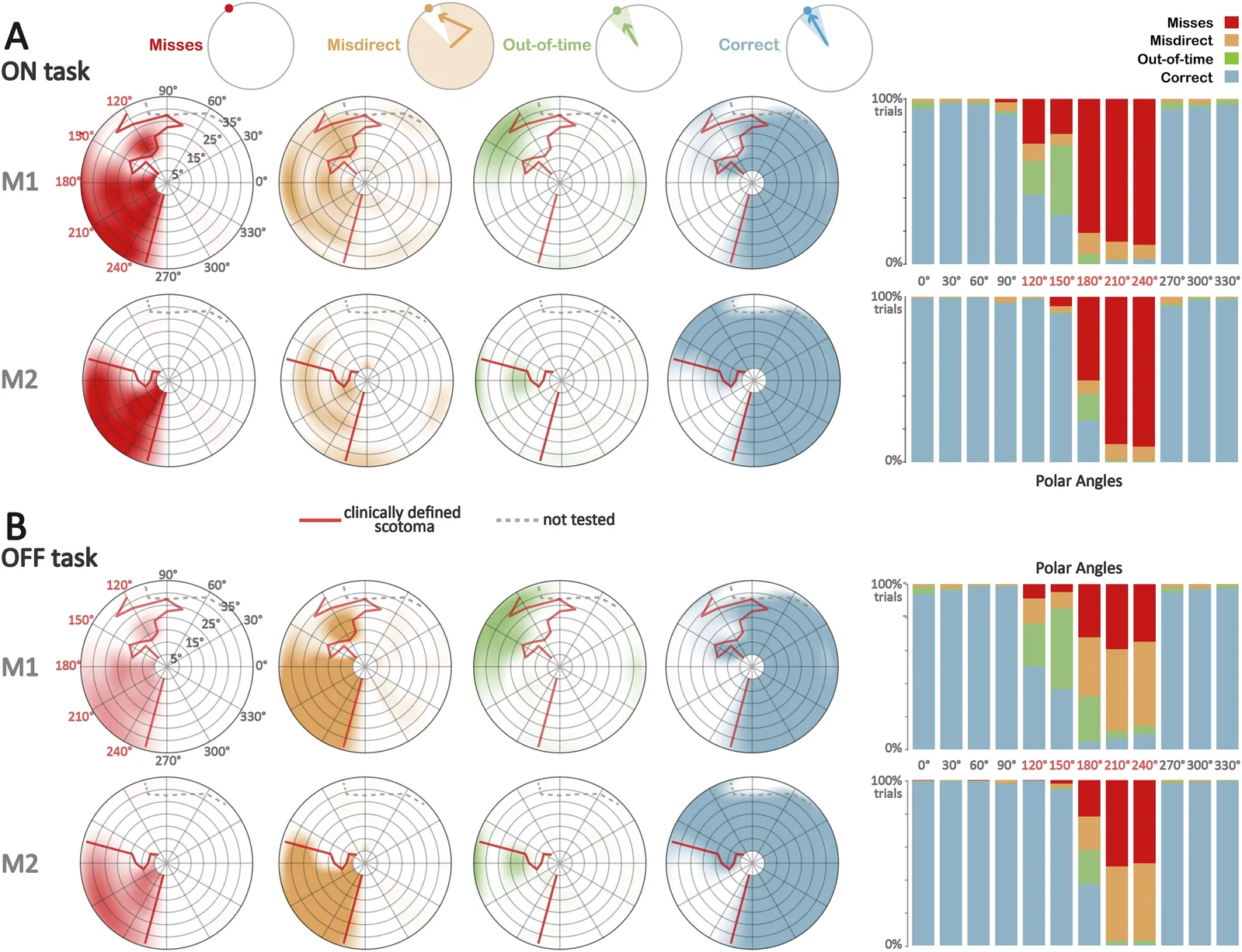
Distribution of four behavioral outcomes across the visual field in the ON (A) and OFF (B) tasks. Ratio of misses (red) misdirects (yellow), out-of-time (green), and correct (blue) responses for M1 (top) and M2 (bottom) are shown either for individual target positions (polar plots, left) or grouped by polar angle (bar plots, right). Small, colored circles above each column schematically illustrate the saccade trajectories (arrows) for each response category, with colored areas indicating the maximum angular deviation from the target (dots). Labels for polar angles representing the contralesional (beyond the vertical meridian) and ipsilesional hemifields are shown in red and black, respectively. Left: Red lines indicate the perimetrically defined scotoma, identified as the region where the ratio of misses exceeded that relative to the misses at mirror locations within the ipsilesional hemifield during the ON task; dotted gray lines indicate visual field locations where eye movements could not be tracked due to experimental constraints (see Methods). For visualization purposes, the data in the polar plots are smoothed using spline interpolation.

### Residual blind-field responses in the fixation-ON task

Although misses dominated fixation-ON performance within the scotoma, target-present trials also elicited two atypical responses: “out-of-time” and “misdirect” (Fig. 3A). The former consisted of direct but delayed saccades that reached toward the target location yet entered the target window only after the 200 ms temporal criterion (Fig. 3A, green). The latter consisted of trials in which the initial saccade deviated from the target vector and could be followed by one or more corrective movements (Fig. 3A, yellow).

#### Post-lesion increase of target-independent saccades

Target-independent saccades in catch trials increased in both animals, indicating that V1 damage altered baseline oculomotor behavior even in the absence of a visual target. In M1, eye movements in catch trials increased from 3.23% prelesion to 6.75% postlesion (*P* < 0.0001, exact binomial test; Fig. S3). In M2, they increased from 0.65% prelesion to 7.5% (*P* < 0.0001).

One plausible interpretation is that, after the V1 lesion, the monkeys adopted a more exploratory oculomotor strategy, shifting gaze toward the blind hemifield to sample whether a target might be present within the scotoma (Arsenault et al. 2019). A related idea has been supported in humans using simulated hemianopia, in which the field defect itself was sufficient to impair visual exploration and trigger spontaneous oculomotor adaptation over time (Schuett et al. 2009). Because such an increase in target-independent saccades could inflate the assessment of postlesion responses, we used the postlesion target-independent eye movement rate in catch trials for each monkey (*p₀*) as a conservative baseline against which to evaluate target-evoked responses in the scotoma.

#### Within-scotoma saccade initiation exceeds the aggregate catch-trial baseline

We next asked whether target-present trials within the scotoma generated saccades more often than expected from target-independent postlesion oculomotor activity. For each scotoma location, the response metric was the proportion of target-present trials on which any saccade was initiated (i.e., either Correct, Out-of-time, or Misdirect). This proportion was compared with the aggregate fixation-ON catch-trial saccade rate, *p₀* (M1: 6.75%; M2: 7.5%; see subsection Directional Analysis of Atypical Saccades and Catch-Trial Saccades). Because catch trials contained no target and therefore no assigned target location, this analysis tests whether target-present saccade initiation exceeds an aggregate, target-independent baseline.

In M1, the probability of observing a saccade in response to targets presented in the scotoma exceeded the aggregate baseline exploratory movements at several locations. Significant effects were concentrated mainly in the upper-left quadrant, with particularly high saccade-initiation probabilities along the 150° meridian at larger eccentricities (e.g., 96.0%, 96.3%, and 75.0% at 25°, 30°, and 35°, respectively; all *P* < 0.0001, FDR-corrected). Additional clusters were present along the 120° meridian (15° to 35° eccentricity) and, to a lesser extent, along the 90°, 180°, and 210° meridians (5° to 30° eccentricity) (Fig. 4; Table S4A and B).

**Figure 4:**
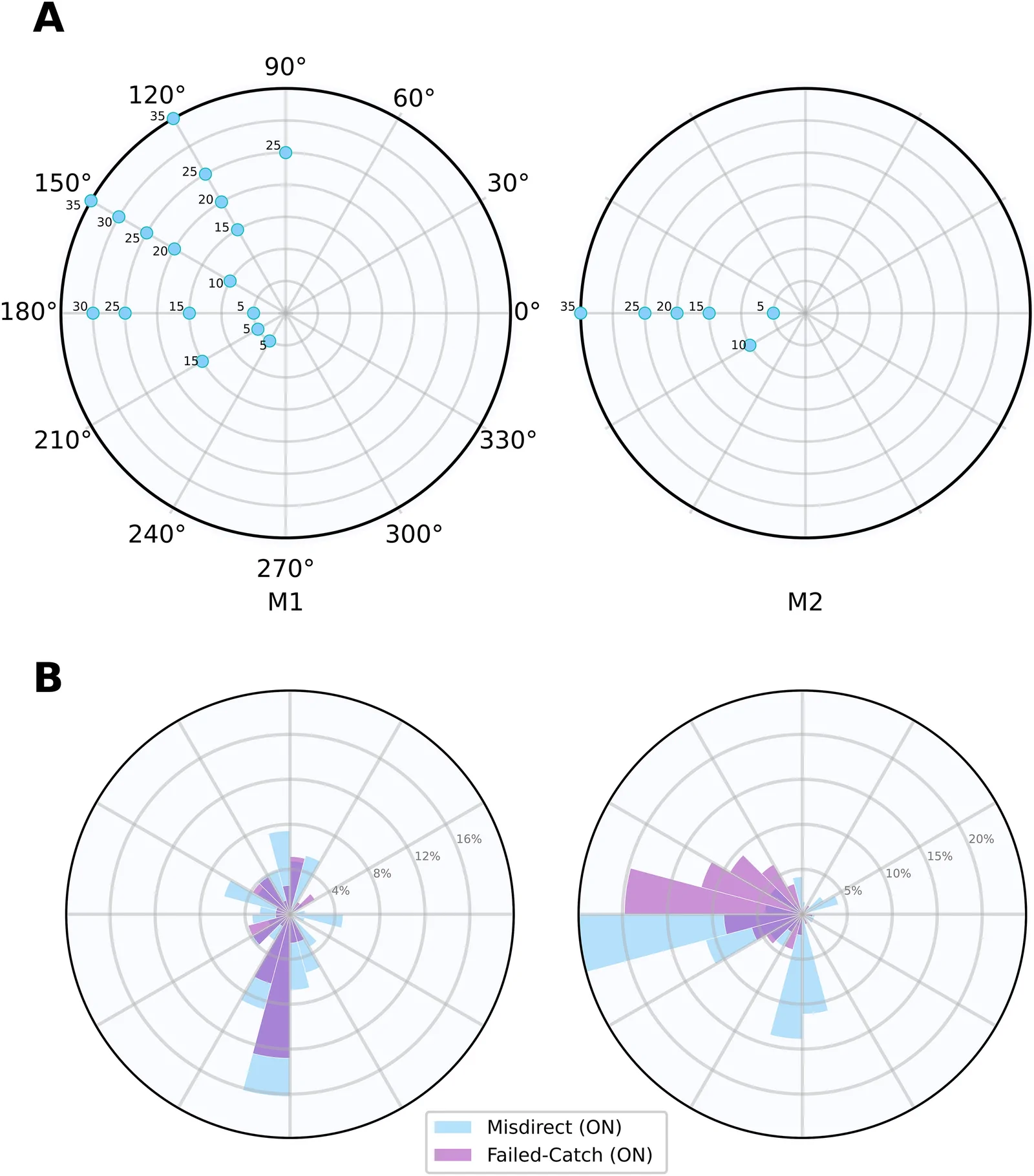
A) Target-present saccades exceed an aggregate catch-trial baseline within the scotoma. Polar plots for M1 and M2 showing only those scotoma locations at which the target-present saccades (Correct + Out-of-time + Misdirect, pooled) occurred significantly more often than postlesion target-independent saccades in fixation-ON catch trials (i.e. aggregate baseline: *p₀*: M1 = 6.75%; M2 = 7.5%). Significance was assessed separately for each target location using a one-sided exact binomial test against *p₀*, with Benjamini–Hochberg FDR correction across scotoma locations (α = 0.05). Numbers adjacent to the symbols indicate eccentricity for significant locations shown. B) Rose diagrams comparing the absolute directions of failed catch-trial saccades and Misdirect responses in the fixation-ON task. Failed catch-trial saccades (purple) occurred when no target was present, and the animal broke fixation. Misdirect responses (blue) occurred on target-present trials in which the first saccadic segment deviated from the target vector. Each bar represents the percentage of trials in a 15° directional bin, normalized independently within each distribution; therefore, bar heights compare directional shape rather than absolute response frequency. The polar-angle convention is the same as in panel A. In M1, the two distributions did not differ significantly (Watson U^2^ = 0.092, BH-FDR *P* = 0.457) and both concentrated near the lower scotoma border (240°). In M2, the two distributions differed significantly (Watson U^2^ = 0.873, BH-FDR *P* = 0.001), but both were non-uniformly distributed and concentrated near the scotoma boundary, indicating that this postlesion spatial preference is present independently of target onset. Scotoma angular ranges: M1, 90° to 240°; M2, 180° to 240°.

In M2, this pattern was limited to a restricted set of scotoma locations. Significant effects were concentrated along the horizontal (180°) meridian, particularly at mid-to-high eccentricities. For example, the proportion of target-present trials that generated a saccade reached 87.8% at 15° and 60.5% at 35° eccentricity (both *P* < 0.0001, FDR-corrected). Additional significant locations were observed at 20° along 180° (57.5%, *P* < 0.0001) and at 10° along 210° (19.5%, *P* < 0.05).

As a control, we repeated this analysis after excluding unrewarded Misdirect saccades from the response metric. The overall spatial pattern remained largely unchanged (Fig. S4), indicating that above-baseline initiation effects were not driven solely by misdirected trajectories. To assess whether Out-of-time responses specifically contributed to this above-baseline pattern, rather than being carried by Misdirect or Correct saccades, we repeated the binomial test using only Out-of-time trials as the numerator, against the same baseline (*p₀*). In M1, Out-of-time generation exceeded *p₀* at six scotoma locations along the 120° and 150° meridians (proportions 36.0 to 81.5%; all p_FDR < 0.001) and was absent along the 240° meridian (0 of 358 trials). Because these locations lie along the upper scotoma border, this target-location dependence cannot be dissociated from a graded decline in sensitivity across that border (subsection Early Residual Visuomotor Behavior, Scotoma-border Bias, and Temporal Cueing); as for the aggregate analysis, this test does not establish target-specific detection (see below).

The global false-alarm rate (*p₀*) is an aggregate, target-independent baseline estimate of postlesion saccade initiation, not a spatially resolved map of false-alarm saccade probability. Accordingly, the significant locations in Fig. 4A identify scotoma sites where target-present trials produced more saccade initiations than expected from the aggregate catch-trial rate. However, they do not, in isolation, provide conclusive evidence of location-specific target detection. Because the directions of catch-trial saccades were themselves non-uniformly distributed across the visual field (Fig. 4B), we next used directional analyses to dissociate target-independent exploratory bias from target-related orienting across the three atypical response classes (subsection Out-of-time Responses may be Target-location-dependent but Overlap Spatially with Scotoma-border Orienting).

#### Out-of-time responses may be target-location-dependent but overlap spatially with scotoma-border orienting

We next asked whether atypical saccades within the operationally defined scotoma reflected trial-specific target direction, target-independent postlesion saccadic activity, or a mixture of both. All analyses in this section refer to the fixation-ON task, where catch trials provided a target-absent benchmark for postlesion saccadic behavior.

Target-present trials at scotoma locations frequently produced atypical responses. Out-of-time responses, defined as direct first saccades that failed to enter the target window within the 200-ms post-fixation-break criterion (subsection Behavioral Outcome Categorization), accounted for 9.0% ± 7.1% of target-present trials, averaged across animals and contralesional target locations. These trials were not rewarded. Misdirect responses, defined by an initial saccade segment deviating from the target vector by ≥15°, were less frequent (3.8% ± 0.4%) and were usually unrewarded. Trials in which a Misdirect first saccade was followed by corrective saccades that ultimately reached the target window were rare (M1: 0.02% of scotoma-target trials, mean n ≈ 1 trial per location, SEM ≈ 0.4; M2: 0.01%, mean *n* < 0.5, SEM ≈ 0.2) and were rewarded only if the full eye-movement sequence still satisfied the task timing and fixation criteria.

Out-of-time responses were not uniformly distributed across the scotoma. They clustered at locations with correct-response rates below 50% and were concentrated in the upper-left quadrant in M1 (81.3% of all Out-of-time responses) and along the horizontal meridian in M2 (80.6%). Misdirect responses showed a similarly structured pattern: 64.6% in M1 and 77.8% in M2 were directed into the contralesional hemifield in which the target had appeared. This coarse hemifield correspondence is consistent with the spatial organization of the scotoma. Still, it does not establish target-specific guidance, because catch-trial saccades were also spatially biased after the lesion.

##### Out-of-time responses emerge where correct saccades decline

To examine target-related orienting and target-independent spatial bias, we analyzed target-present responses in target-relative space, expressing each saccade direction as its circular deviation from the trial-specific target polar angle. Catch-trial saccades, which have no trial-specific target vector, were retained for comparison in absolute directional space.

Out-of-time saccade directions in target-relative space were highly concentrated near 0° in both animals (M1: $ {\bar{R}} $ = 0.998, mean direction = 359.3°, Rayleigh *P* < 0.001, Rao *T* = 335.2, Rao *P* ≤ 0.001; M2: $ {\bar{R}} $ = 0.999, mean direction = 1.2°, Rayleigh *P* < 0.001, Rao *T* = 339.4, Rao *P* ≤ 0.001). The median absolute deviation from the target polar angle was 1.8° (IQR 0.6° to 3.4°; mean ± SD: 2.6° ± 3.2°) in M1 and 1.4° (IQR 1.2° to 2.5°; mean 2.0° ± 1.6°) in M2. Out-of-time saccade directions also differed significantly from catch-trial saccade directions in absolute deviational space (Watson *U^2^*: M1 = 1.258, FDR *P* = 0.001; M2 = 0.790, FDR *P* = 0.001) and from misdirected saccade directions in absolute deviational space (Watson U^2^: M1 = 2.977, FDR *P* = 0.001; M2 = 0.588, FDR *P* = 0.001). The absolute deviation of out-of-time saccades from the target polar angle was smaller than that of misdirected saccades in both animals (Mann–Whitney U: M1 = 356.0, *P* < 0.001, r = 0.973; M2 = 91.0, *P* < 0.001, r = 0.935). Misdirected saccade directions were non-uniform in absolute directional space (Rayleigh and Rao’s spacing tests, all *P* ≤ 0.001 in both animals). Thus, Out-of-time responses were directionally distinct from spontaneous catch-trial saccades. Because a directional criterion defines the category, their target-relative concentration cannot by itself demonstrate target attraction; however, their frequency varied systematically with target location (subsection Within-scotoma Saccade Initiation Exceeds the Aggregate Catch-trial Baseline and below), and they were concentrated at intermediate eccentricities where correct responses declined, consistent with a graded transition from accurate to atypical responding across the scotoma.

##### Catch-trial and Misdirect saccades share a scotoma-border-directed spatial bias

We next examined whether Misdirect responses overlapped with the target-independent saccades observed on catch trials. This analysis was performed in absolute directional space so that catch-trial and target-present Misdirect saccades could be compared in the same visual-field coordinates.

In M1, both the catch-trial and Misdirect saccade distributions were better described by a two-component von Mises mixture model than by a unimodal model (catch: ΔBIC = 12.5; misdirect: ΔBIC = 31.3; see subsection Directional Analysis of Atypical Saccades and Catch-Trial Saccades). Catch-trial saccades showed two components: a concentrated mode near the lower scotoma border (254°, κ = 6.64) and a more diffuse mode near the upper scotoma border (114°, κ = 1.80). Misdirect saccades showed a similar bimodal structure: a highly concentrated mode near the lower scotoma border (267°, κ = 13.61) and a diffuse mode in the upper-left quadrant (128°, κ = 0.67). The two distributions did not differ significantly in absolute directional space (Watson *U^2^* = 0.092, FDR *P* = 0.457), consistent with substantial overlap between Misdirect responses and target-independent scotoma-border orienting in M1.

In M2, the catch-trial distribution was best described by a unimodal model (ΔBIC = −0.7), with a single mode centered at 171°, just above the upper scotoma border at 180°. The Misdirect distribution in M2 was bimodal (ΔBIC = 12.4), with a dominant mode at 188° (mixing weight 0.73, κ = 97.85), located just below the upper scotoma border, and a secondary diffuse mode at 234°. Unlike M1, the catch-trial and Misdirect distributions differed significantly in M2 (Watson *U^2^* = 0.873, FDR *P* = 0.001). However, both distributions remained concentrated near scotoma-border angles, indicating that even when Misdirect responses differed from catch-trial saccades, they were still shaped by the same broad border-related spatial bias.

As a robustness check, the directional analysis was repeated for Out-of-time and Misdirect saccades using the full-segment direction estimator (subsection Directional Analysis of Atypical Saccades and Catch-Trial Saccades). Results were consistent for Out-of-time saccades in both animals ($ {\bar{R}} $ ≥ 0.988 under both methods; mean directions within 1.5°). For Misdirect saccades, results were also consistent in M2 (Watson *U^2^* Misdirect vs. catch: full-segment FDR *P* = 0.001, primary 50 ms estimator FDR *P* = 0.001). In M1, however, the full-segment method produced a significant difference between Misdirect and catch-trial distributions (Watson *U^2^* = 0.257, BH-FDR *P* = 0.013) that was not present under the primary 50-ms estimator (*U^2^* = 0.092, *P* = 0.457). This sensitivity to the direction-estimation method further supports treating Misdirect responses, especially in M1, as a mixed category rather than as a clean measure of target-specific residual vision.

##### The border bias is broadly expressed in both animals but spatially more specific in M2

To quantify the directional structure of catch-trial and Misdirect saccades relative to the scotoma, we compared observed saccade-direction proportions with the geometric null across three nested spatial zones surrounding the scotoma anchor angles (subsection Directional Analysis of Atypical Saccades and Catch-Trial Saccades). At the broad-border zone (±30°), the proportion of saccades directed toward the scotoma-border region exceeded the geometric null in both distributions and both animals (M1 catch: 84.4% vs. 58.6% null, binomial *P* = 0.0003; M1 Misdirect: 75.0% vs. 58.6%, *P* < 0.001; M2 catch: 70.8% vs. 33.3%, *P* < 0.001; M2 Misdirect: 66.7% vs. 33.3%, *P* < 0.001; all BH-FDR corrected) (Fig. 4B). Broad scotoma-border overrepresentation was therefore present even when no target appeared, and Misdirect responses on target-present trials also expressed it.

In M2, this bias remained significant at finer spatial scales. The proportion of saccades directed toward predefined scotoma-related angular zones exceeded the geometric null both within ±7.5° of scotoma anchor angles (catch: 38.9% vs. 20.8% null, *P* < 0.001; Misdirect: 48.6% vs. 20.8%, *P* < 0.001) and within ±15° (catch: 54.2% vs. 25.0%, *P* < 0.001; Misdirect: 51.4% vs. 25.0%, *P* < 0.001; all BH-FDR corrected). The Misdirect and catch-trial distributions in M2 differed (Watson *U^2^* = 0.873, FDR *P* = 0.001) (Fig. 4B), with the dominant Misdirect mode at 188° (just below the upper scotoma border) compared with the catch-trial mode at 171° (just above it).

In M1, the proportion of saccades directed toward the scotoma did not exceed the geometric null at finer spatial scales, within ±7.5° (catch: 28.9% vs. 26.7%, *P* = 0.42; Misdirect: 19.6% vs. 26.7%, *P* = 0.98) or within ±15° (catch: 62.2% vs. 50.3%, *P* = 0.073; Misdirect: 53.4% vs. 50.3%, *P* = 0.25; all BH-FDR corrected). The Misdirect and catch-trial distributions did not differ in absolute directional space (Watson *U^2^* = 0.092, FDR *P* = 0.457), consistent with their shared bimodal structure, which is concentrated near both scotoma borders. Thus, in M1, the Misdirect distribution was especially compatible with target-independent scotoma-border orienting.

The above-baseline locations in Fig. 4A and the directional preferences of catch-trial saccades showed different degrees of spatial correspondence across animals. In M2, the Fig. 4A locations clustered along the 180° meridian, and the dominant mode of catch-trial saccades was at 171°, within 9° of the upper scotoma borders. Above-baseline target-present saccade initiation and target-independent border-directed orienting were therefore spatially close, reinforcing the need to interpret Fig. 4A together with the directional analyses. In M1, the locations at which target-present saccade initiation exceeded the aggregate catch-trial baseline were distributed across the upper-left quadrant (120° to 150° meridians). In contrast, catch-trial saccades concentrated near the lower scotoma border (dominant mode at 254°).

##### Directional structure across atypical response classes

Taken together, the directional analyses reveal partially separable components of early postlesion oculomotor behavior. Out-of-time responses were direct but delayed or incomplete saccades whose generation depended on target location. They coincided with a decline in correct responses across the same region, consistent with a graded transition rather than a spatially uniform bias. Catch-trial saccades revealed a target-independent bias toward scotoma-border regions. Misdirect responses occupied an intermediate position, showing weaker target-relative structure and, in absolute coordinates, overlapping substantially with the catch-trial border bias, particularly in M1. These classes are better understood as successive stages of a degraded response profile, in which accurate saccades give way to directionally appropriate but temporally deficient responses, then to misdirected and ultimately absent responses as sensitivity declines across the scotoma.

### Temporal cueing in the fixation-OFF task enhances blind-field saccades

Here, we examined how responses in the scotoma changed in the fixation-OFF task, in which fixation offset signaled target presentation. Across all contralesional target locations, atypical responses increased in the fixation-OFF compared with the fixation-ON condition. Out-of-time and misdirect responses accounted for 13.9% ± 11.7% and 14.4% ± 1.7% of trials, respectively (i.e., ~30% of all contralesional trials, compared with ~15% in fixation-ON).

#### *Pooled comparison of fixation-ON* vs. *fixation-OFF responses within the scotoma*

To quantify the effect of temporal cueing specifically within perimetrically blind regions, we compared fixation-ON and fixation-OFF responses at contralesional target locations for which the fixation-ON task yielded no correct response. This conservative approach minimized the potential contribution of partially spared visual patches within the scotoma. For each monkey, we then used bootstrap resampling (see Methods) to compare the proportions of the four response categories (Correct, Out-of-time, Misdirect, Miss) between ON and OFF conditions (Fig. 5A).

**Figure 5:**
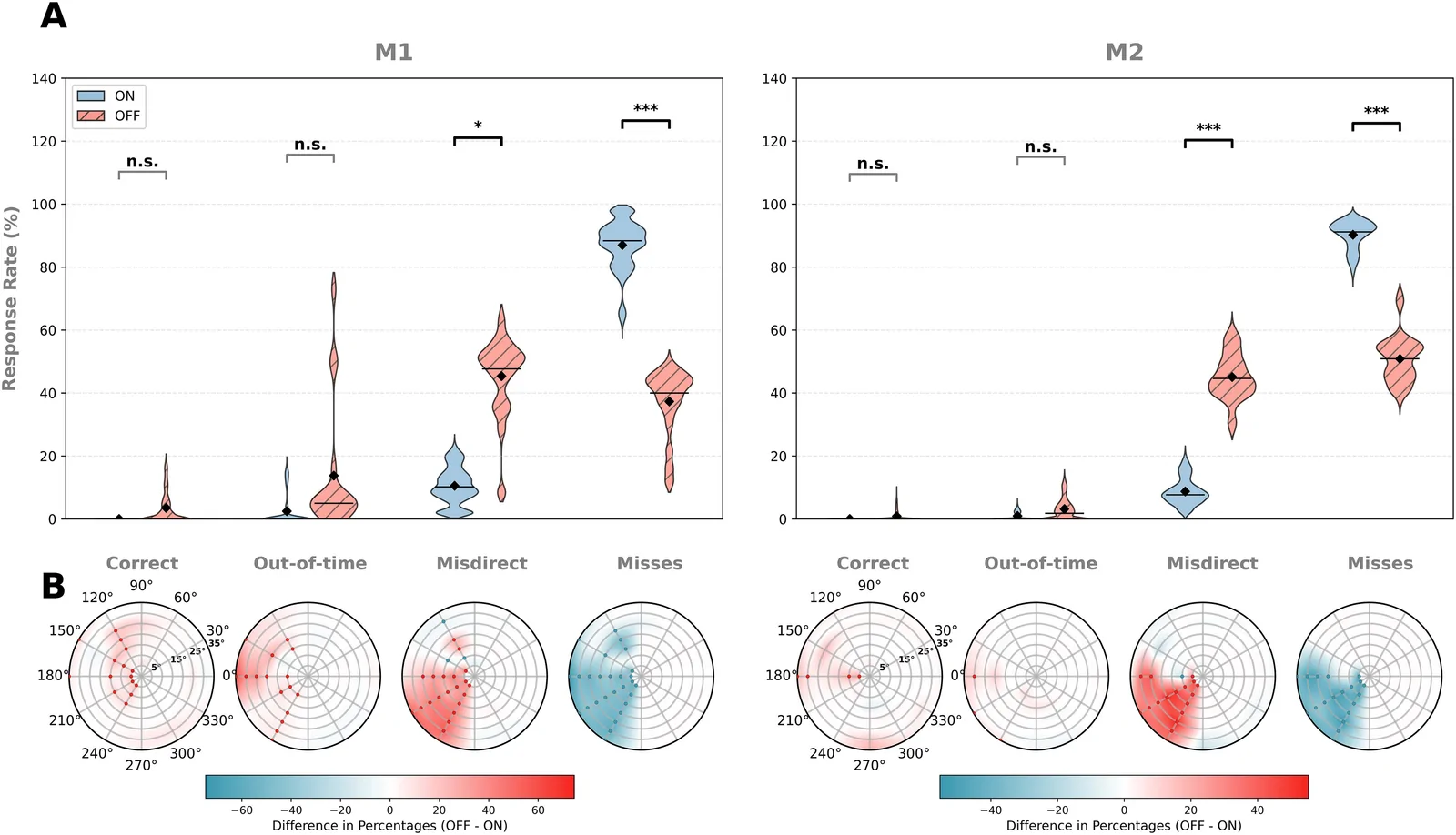
Temporal cueing in the fixation-OFF task shifts blind-field responses from misses to misdirected saccades. A) Violin plots for M1 (left) and M2 (right) show the distribution of response percentages across scotoma target locations that yielded no correct responses in the fixation-ON task, separated by Correct, Out-of-time, Misdirect, and Misses. Blue = fixation-ON; red = fixation-OFF. Violin width indicates the across-target distribution; black diamonds mark the mean, and horizontal black lines the median. Brackets indicate the significance of the OFF vs. ON comparison for each response category (n.s., not significant; ^*^P < 0.05; ^**^P < 0.001; two-sided bootstrap sign test, FDR-corrected). At the pooled level, fixation-OFF produced a marked increase in misdirect responses and a corresponding decrease in misses, whereas correct and out-of-time responses did not differ significantly. B) Polar maps show the OFF − ON difference in response percentage for each category at each target location within the perimetrically defined scotoma (red, OFF > ON; blue, ON > OFF). Colored dots mark locations with significant OFF–ON differences after two-sided bootstrap testing and FDR correction. Concentric rings indicate eccentricity (5° to 35°) and radial lines indicate polar angle. OFF-related increases in misdirect responses were widespread across the scotoma and were mirrored by complementary decreases in misses. In contrast, OFF-related increases in correct and out-of-time responses were spatially restricted, occurring at a limited subset of locations, particularly near scotoma borders.

In M1, temporal cueing produced no significant pooled increase in correct responses (ON median = 0.0%, OFF median = 1.7%; 95% CI [0.00, 21.54]%; *P* = 0.4629), and no significant pooled increase in out-of-time responses (ON median = 0.0%, OFF median = 4.9%; 95% CI [0.00, 61.86]%; *P* = 0.2112). In M2, the same pattern was observed: correct responses did not differ significantly between tasks (ON median = 0.0%, OFF median = 0.0%; 95% CI [0.00, 7.41]%; *P* = 0.7925) nor did out-of-time responses (ON median = 0.0%, OFF median = 1.8%; 95% CI [−5.13, 12.96]%; *P* = 0.6661).

By contrast, misdirected responses increased significantly in the fixation-OFF task in both animals. In M1, misdirected responses increased from 10.0% to 47.7% (Δ_median ≈ 37.7%, *P* = 0.0144), while in M2 they rose from 7.7% to 45.5% (Δ_median ≈ 37.8%, *P* = 0.0005). These increases were mirrored by complementary decreases in misses (M1: from 88.6% to 38.3%, Δ_median = −50.3%, *P* < 0.0001; M2: from 90.9% to 50.9%, Δ_median = −40.0%, *P* = 0.0003) (Table 1). Therefore, at the pooled spatial level, the main effect of temporal cueing was not to convert misses into correct responses, but rather to convert misses into misdirected saccades.

**Table 1:** Bootstrap comparison of fixation-OFF and fixation-ON responses in the scotoma. For each monkey and response category, the table reports median percentages, the standardized effect size *t*-scores (*t* = mean(Δ)/SE(Δ), where Δ is the bootstrap OFF − ON difference), the associated two-sided *P*-value, and the 95% confidence interval for the OFF–ON difference. All statistics were computed from 1,000 bootstrap iterations, pooling targets within the scotoma that yielded no correct response in the fixation-ON task. Significant differences (*P* < 0.05) indicate a reliable difference in the proportion of that response category between OFF and ON conditions.

Subject	Correct	Out-of-time	Misdirect	Misses
M1	ON median = 0.0% OFF median = 1.7% *t* = 0.74, *P* = 0.4629 CI = [0.00, 21.54]	ON median = 0.0% OFF median = 4.9% *t* = 0.67, *P* = 0.2112 CI = [0.00, 61.86]	ON median = 10.0% OFF median = 47.7% *t* = 2.25, ***P* = 0.0144** CI = [3.77, 60.66]	ON median = 88.6% OFF median = 38.3% *t* = −4.22, ***P* < 0.0001**, CI = [−75.41, −27.30]
M2	ON median = 0.0% OFF median = 0.0% *t* = 0.42, *P* = 0.7925 CI = [0.00, 7.41]	ON median = 0.0% OFF median = 1.8% *t* = 0.51, *P* = 0.6661, CI = [−5.13, 12.96]	ON median = 7.7% OFF median = 45.5% *t* = 3.38, ***P* = 0.0005** CI = [15.65, 57.67]	ON median = 90.9% OFF median = 50.9% *t* = − 3.51, ***P* = 0.0003** CI = [−59.81, −16.53]

Bold values indicate statistically significant OFF–ON differences (*p* < 0.05, two-sided bootstrap sign test, FDR-corrected).

These ON–OFF differences were absent before the lesion. In fact, the same target locations showed no significant differences between fixation-ON and fixation-OFF for any response category (Table 2). Likewise, the ON–OFF comparison performed on ipsilesional target locations yielded negligible changes in response-class proportions (Fig. S5), consistent with the interpretation that OFF-related changes in the contralesional field reflect genuine postlesion dynamics of eye movements in the blind field, rather than generic task effects.

**Table 2:** Bootstrap comparison of fixation-OFF and fixation-ON responses at the same target locations prior to V1 lesion. For each monkey and response category, the table reports median percentages, the standardized effect size *t*-scores (*t* = mean(Δ)/SE(Δ), where Δ is the bootstrap OFF − ON difference), the associated two-sided *P*-value, and the 95% confidence interval for the OFF–ON difference. All statistics were computed from 1,000 bootstrap iterations, using prelesion data from contralesional target locations that were later classified as belonging to the scotoma and pooling targets within the scotoma that yielded no correct responses in the fixation-ON task. The absence of significant ON–OFF differences before the lesion indicates that the task dissociations observed postlesion were not present in the intact state.

Subject	Correct	Out-of-time	Misdirect	Misses
M1	ON median = 98.8% OFF median = 100.0% *t* = 0.08, *P* = 0.9421 CI = [−7.41, 7.59]	ON median = 0.0% OFF median = 0.0% *t* = 0.04, *P* = 0.9676 CI = [−5.06, 5.33]	ON median = 0.0% OFF median = 0.0% *t* = −0.14, *P* = 0.9212 CI = [−7.10, 5.19]	ON median = 0.0% OFF median = 0.0% *t* = nan, *P* = 1.0000 CI = [0.00, 0.00]
M2	ON median = 100.0% OFF median = 100.0% *t* = 0.22, *P* = 0.8192 CI = [−4.40, 6.42]	ON median = 0.0% OFF median = 0.0% *t* = −0.24, *P* = 0.7418 CI = [−4.81, 4.40]	ON median = 0.0% OFF median = 0.0% *t* = −0.03, *P* = 0.9795 CI = [−2.75, 2.35]	ON median = 0.0%, OFF median = 0.0% *t* = nan, *P* = 1.0000, CI = [0.00, 0.00]

#### Target-specific ON–OFF differences reveal spatially specific cueing effects

Although the pooled analysis showed that temporal cueing primarily increased misdirect responses, target-specific analyses revealed a more differentiated spatial pattern. At individual scotoma locations, fixation-OFF also produced significant increases in correct and out-of-time responses relative to fixation-ON, but these effects were spatially restricted to a subset of blind-field locations and, therefore, nonsignificant in the pooled analysis (Fig. 5B; Figs. S1–S4; Tables S1–S3).

For correct saccades, OFF-related improvements were primarily localized to mid-peripheral targets and near the borders of the scotoma. In M1, significant gains were observed both near the upper and lower borders of the blind field, and at mid-peripheral locations within more densely impaired regions. In M2, the increase in correct responses was more limited and was largely confined to locations along the left horizontal meridian at 5°, 10°, 30°, and 35° eccentricity (Fig. 5B).

For out-of-time responses, M1 showed a relatively broader distribution of significant OFF-related improvements, spanning both mid- and far-peripheral sites at 120°, 150°, 180°, 210°, and 240° polar angles. In M2, significant increases in out-of-time responses were sparse and limited to far-peripheral locations, at 30° eccentricity at 180° and 35° eccentricity at 210° polar angle.

Predictably, the effect of fixation-OFF on misdirect saccades was robust and widespread. In M1, significant increases in misdirect responses were distributed across multiple sites in the blind field and were systematically paired with reductions in misses at corresponding locations. M2 displayed a similar pattern, with OFF-related increases in misdirect responses extending across large portions of the scotoma.

Overall, these target-specific analyses refine the pooled comparison in two important aspects. First, they show that temporal cueing can, at some specific scotoma locations, enhance not only misdirected responses but also correct and out-of-time saccades. Second, they indicate that these improvements remain spatially limited, while the dominant effect of the fixation-OFF paradigm shortly after V1 damage was to produce a broad shift from misses to misdirected responses, where the initial eye movement remains inaccurately directed.

## Discussion

Using wide-field mapping in macaques tested only 3 to 4 weeks after unilateral V1 lesion, we show that blind-field stimuli can still elicit structured visuomotor responses well before the chronic blindsight stage usually examined in nonhuman primates. Across the scotoma, these residual responses were expressed predominantly as delayed or initially misdirected saccades, and temporal cueing in the fixation-OFF task mainly converted misses into such atypical responses rather than into fully correct saccades, as previously reported in chronic stages (Mohler and Wurtz 1977; Cowey and Stoerig 1995; Moore et al. 1995; Schmid et al. 2010; Isa and Yoshida 2021).

### Early residual visuomotor behavior, scotoma-border bias, and temporal cueing

A first relevant result is that residual blind-field visuomotor behavior is already measurable in the early postlesion phase, provided that it is assessed with sufficient spatial density and with response categories that go beyond a dichotomous correct vs. miss score. Both monkeys executed direction-specific saccades toward the border of the scotoma during catch trials in which no targets were presented. This indicates that they exhibit adaptive exploratory behavior directed at the scotoma borders, not triggered by visual stimuli within the scotoma. Moreover, even under our conservative, perimetry-like definition of the scotoma, requiring both increased misses and reduced correct responses, target-present trials within the blind field could still produce saccades in the fixation-ON task. These responses were sparse but spatially structured rather than randomly distributed across the blind field. The above-baseline effects (Fig. 4A) should nevertheless be interpreted conservatively. They identify locations where target-present trials produced more saccade initiations than expected from the aggregate catch-trial baseline, not locations where target-specific detection is proven independently of spatial false-alarm bias. This pattern converges with recent human evidence that the subacute period after occipital stroke is not merely a transition toward chronic blindness (Saionz et al. 2020).

#### Compensatory scotoma-border orienting is part of the early phenotype

After the V1 lesion, the rate of target-independent saccades on catch trials increased relative to the prelesion baseline (Table 2). This behavior likely reflects a more active sampling strategy, in which the animals intermittently shift gaze toward the affected field to test whether a target is present and a reward can be obtained. Critically, catch-trial saccades were themselves directionally biased toward scotoma borders despite the absence of any peripheral target. This indicates that compensatory orienting toward the scotoma borders occurred independently of target presentation and should be considered part of the early postlesion oculomotor phenotype rather than mere noise. However, the directional analyses indicate that this target-independent border bias does not fully account for all blind-field target-present responses. This is especially clear for Out-of-time responses, whose generation depended on target location in a way not explained by amplitude, though their concentration at the upper border does not isolate them from border-related sensitivity.

The directional analyses of Out-of-time, Misdirect, and catch-trial saccades reveal partially separable aspects of early postlesion oculomotor behavior. The first is a target-independent scotoma-border bias. At the broad border zone (±30°), the proportion of saccades directed toward the scotoma boundary exceeded the geometric null in both catch-trial and Misdirect saccade distributions in both animals, regardless of whether a target appeared on a given trial. Whether this border-directed preference reflects active spatial sampling, reward-seeking probing of the scotoma boundary, residual sensitivity along a graded scotoma border, or another postlesion oculomotor reorganization cannot be determined from the present data. The two animals differed in the spatial specificity of this scotoma-border bias: in M2, significant overrepresentation was present at all three spatial scales examined, including the most internal part of the scotoma; in M1, significance was restricted to the broad border zone.

In this context, the term “scotoma border” should not be taken to imply a sharp transition from intact to absent sensitivity. Although our perimetric criterion yields a discrete, operationally defined boundary (based on the miss/correct threshold), this operational sharpness does not imply an equally sharp physiological transition. A graded decline in sensitivity across the border region is more plausible, and residual sensitivity along this gradient may itself contribute to the observed bias of saccades toward border-adjacent locations relative to more deeply affected regions.

#### Out-of-time responses mark a graded transition rather than isolated target-directed vision

The second component is target-location dependence, most evident for Out-of-time responses. Although these saccades were infrequent (Fig. 3) and unrewarded, their generation varied systematically with target position: within M1’s scotoma, Out-of-time responses exceeded the aggregate catch-trial baseline at locations along the 120° and 150° meridians and were absent along 240° (subsection Within-scotoma Saccade Initiation Exceeds the Aggregate Catch-trial Baseline), a pattern not explained by saccade amplitude, since it persisted at matched eccentricity. Their target-relative concentration ($ {\bar{R}} $ ≥ 0.998) is, in part, a consequence of the directional criterion defining the category and does not by itself establish attraction to the specific target location. We therefore interpret Out-of-time responses as target-location–dependent rather than as direct evidence of target-specific residual vision. Two features constrain this interpretation. First, the significant locations lay along the upper scotoma border, so target-location dependence cannot be separated from a graded decline in sensitivity across that border. Second, the restriction of the effect to the upper border, despite the lower border (240°) being equally adjacent to the intact field, was not accounted for by the present analyses and may reflect retinotopic asymmetries of the lesion.

#### Misdirect responses as a mixed phenotype

Misdirect responses occupied an intermediate position. They showed some target-relative structure, but their absolute directional distributions also overlapped with catch-trial saccades, especially in M1. We therefore interpret Misdirect responses cautiously, as a mixed phenotype in which weak target-related influences and compensatory scotoma-border orienting may contribute in different proportions across animals and locations, rather than as unambiguous evidence of target-specific residual vision. In M2, Misdirect responses were slightly more spatially separated from catch-trial saccades. In M1, by contrast, Misdirect and catch-trial distributions were more similar, consistent with a larger contribution of target-independent border-directed orienting. The spatial relationship between Out-of-time responses and catch-trial preferences mirrored this pattern: in M2, all three response classes spatially converged near the 180° meridian, whereas in M1, target-location-dependent Out-of-time responses concentrated in the upper-left quadrant (120° to 150°) while catch-trial and Misdirect saccades concentrated near the lower scotoma border.

#### Temporal cueing increases blind-field saccade initiation

The comparison between fixation-ON and fixation-OFF paradigms revealed a further, task-dependent dissociation. Consistent with classic reports of macaque blindsight in chronic stages (Moore et al. 1995), temporal cueing in the fixation-OFF task increased the expression of blind-field saccades. This effect must, however, be interpreted with caution because catch trials were not included in the fixation-OFF condition, and the increase due to temporal cueing was not uniform across response classes. In the acute postlesion phase, temporal cueing appears to recruit residual blind-field visuomotor processing primarily at the level of saccade initiation and coarse orienting, with more limited benefits for precise target localization. The fixation-OFF task, therefore, amplified the expression of residual or compensatory visuomotor behavior that was already weakly expressed and more spatially constrained in the fixation-ON task. Under this framework, the fixation-ON paradigm identifies a restricted set of blind-field locations at which residual visual signals or target-related biases are strong enough to trigger saccade initiation even without temporal cueing. In contrast, the fixation-OFF task expands this set by reducing temporal uncertainty.

#### An early, degraded phenotype distinct from chronic macaque blindsight

Overall, these findings bear on the long-standing question of whether blindsight reflects the functions of V1-independent pathways that are already active in the intact brain, postlesion adaptive behavior and plasticity, or an interaction between the two (Tamietto and Morrone 2016; Fox et al. 2020). Our data are most consistent with a hybrid account. The presence of structured scotoma-border biases and target-location–dependent out-of-time responses within 3 to 4 weeks of the lesion is consistent with V1-independent visuomotor processing being available very early, before prolonged retraining or large-scale reorganization. Our data cannot fully separate residual visual signals from compensatory oculomotor strategies. This is consistent with the long-standing observation that extrastriate cortex carries V1-independent visual responses on a fast timescale (Girard et al. 1991, 1992). Accordingly, the response profile we observed differs from that typically described at more chronic stages, where the task context can produce a sharper transition from misses to correct saccades (Moore et al. 1995; Yoshida and Isa 2015). Here, the temporal cueing shifted behavior primarily from misses to atypical saccade responses, rather than to fully correct saccades. Stable, accurate saccadic guidance across the scotoma may therefore require substantial postlesion adaptations that develop over weeks to months and are evident in chronic blindsight studies (Moore et al. 1995; Yoshida and Isa 2015; Kaas and Lyon 2018).

### Parsing atypical saccades by response stage

Neurophysiological studies have parsed visually guided saccades into sequential and partially dissociable stages, including early detection, evidence accumulation/decision, target selection, planning, initiation, and trajectory control (Dorris et al. 1997; Schall and Thompson 1999; Supèr et al. 2001; McPeek et al. 2003; Gold and Shadlen 2007). Treating atypical saccades as behaviorally informative readouts of partially preserved visuomotor processing allowed us to distinguish several response phenotypes within the scotoma.

Misses likely reflect failures of visual registration and/or a failure to drive the initiation mechanism above threshold. Out-of-time responses indicate that a saccade was initiated in an appropriate general direction but not completed within the temporal criterion; whether this reflects target-specific guidance or the location-dependence documented above cannot be fully separated. Misdirect responses indicate that target presentation may still influence movement initiation, but that the initial saccade vector was specified only coarsely or inaccurately. However, Misdirect responses should not be interpreted as a pure readout of target-specific processing, because the directional analyses showed that they can overlap with target-independent scotoma-border orienting. Correct responses, in turn, mark timely acquisition of the target location and the successful completion of the entire sequence from target registration to stable fixation.

#### Out-of-time responses are target-location-dependent but temporally deficient

The directional analysis further characterizes these response types. Out-of-time saccades depended systematically on target location in both animals (subsection Within-scotoma Saccade Initiation Exceeds the Aggregate Catch-trial Baseline). Misdirect responses, by contrast, showed substantially weaker and more dispersed target-relative structure, consistent with their interpretation as a mixed category rather than a clean readout of target direction. Within the saccade-stage framework outlined above, this pattern is consistent with a dissociation between the processes that trigger a saccade and those that ensure its timely completion: Out-of-time responses reach initiation but fail the temporal criterion. Whether the direction of these saccades reflects accurate target selection or is partly a consequence of their classification cannot be resolved here. Alternative explanations, including reward-seeking probing of the scotoma border and compensatory scanning strategies, may contribute specifically to the Misdirect response category and cannot be excluded from the present data. In contrast, the Out-of-time category is less readily explained by these alternatives, given that its occurrence varied with target location in a way not fully predictable by a spatially fixed border bias, though its concentration at the upper border prevents full dissociation from border-related sensitivity.

#### Alternative non-target-specific accounts and their limits

Two additional alternative explanations warrant consideration. First, through repeated experience after the lesion, animals may have learned that saccades toward the scotoma border, or to immediately adjacent intact-field regions, carried a relatively higher probability of reward than saccades deeper into the scotoma. Such learning could bias saccade direction toward that region independently of any target-specific visual signal. Under this interpretation, the directional structure observed in Misdirect and catch-trial saccades would reflect learned reward-probability mapping rather than spontaneous spatial orienting or residual visual processing. This possibility is compatible with the catch-trial data, because a learned border-directed strategy could be expressed even on target-absent trials.

Second, small eye movements occurring between fixation break and subsequent target acquisition may have shifted the target into intact visual field regions, thereby enabling later saccades to be guided via the intact geniculo-striate pathway. This mechanism could generate apparently target-directed responses without requiring any residual processing within the scotoma itself. This concern motivated our analytic decision to classify trials according to the first saccadic segment and to retain trials with an initially misdirected first saccade in the Misdirect category, even when subsequent corrective movements brought gaze into the target window. The present data cannot exclude either of these non-target-specific accounts for all atypical responses.

#### Fixation-ON/OFF dissociation in the saccade-stage framework

This response-category decomposition helps explain the fixation-ON/fixation-OFF dissociation (Danckert and Rossetti 2005). The offset of the central fixation reduces temporal uncertainty and increases the probability that weak residual visual signals will trigger a saccade. In our data, this increase mainly reflects atypical responses, especially Misdirect responses, rather than a restoration of consistently correct target acquisition. Thus, temporal cueing facilitates the transition from no saccade to an initiated saccade, but, by itself, does not restore accurate vector specification or reliable target acquisition. This interpretation is consistent with previous macaque work showing that, after V1 lesion, visually guided saccades become less accurate, straighter, and less deliberately initiated (Yoshida et al. 2008; Isa and Yoshida, 2009). The present study extends those observations to the early postlesion period and across a broader extent of the visual field. In this phase, whatever signals influence saccade initiation, whether residual visual processing, reward-probability biases, or scotoma-boundary-related strategies, are sufficient to trigger saccades on a subset of trials, but not to reliably support accurate and timely target localization.

### Candidate neuronal mechanisms

#### Superior colliculus and fixation-OFF disinhibition

The cueing-related increase in Misdirect responses observed in the fixation-OFF task is compatible with a major contribution of the superior colliculus (SC). The SC receives direct retinal input and contributes critically to residual visuomotor function after V1 lesion (Mohler and Wurtz 1977; Moore et al. 1995; Cavanaugh et al. 2016; Tamietto and Morrone 2016; Berman et al. 2017; Basso et al. 2021; Isa et al. 2021). Moreover, in gap-like paradigms, fixation offset disinhibits SC neurons, enhances pre-saccadic buildup, and facilitates movement initiation (Dorris et al. 1997). This mechanism fits the present observation that temporal cueing primarily increased atypical saccade initiation rather than restoring fully correct target acquisition. Recent studies on SC organization may help sharpen this translational bridge, given a new connectivity-based parcellation of the human superior colliculus (Diano et al. 2025) and recent fMRI evidence for lateralized visuotopic organization in the macaque superior colliculus (Sepe et al. 2025).

#### Cortical pathways for finer spatial information

In contrast, the smaller and spatially restricted OFF-related gains in Correct and Out-of-time responses are more consistent with pathways capable of conveying finer spatial information. Candidate substrates include direct geniculo-extrastriate inputs to MT and SC-pulvinar-extrastriate routes. In macaques, a direct projection from the lateral geniculate nucleus (LGN) to middle temporal area (MT) has been demonstrated anatomically (Sincich et al. 2004) and remains functionally active after V1 damage (Schmid et al. 2010). In humans, blindsight functions have been associated with both preserved geniculo-extrastriate connectivity (Ajina, Pestilli, et al. 2015) and pulvinar-extrastriate projections (Kletenik et al. 2022; Bridge, Leopold, and Bourne, 2016). Extrastriate contrast responses are also abnormal but still measurable (Ajina, Rees, et al. 2015), and spared perilesional V1 activity underlies training-induced recovery in patients with cortical blindness (Barbot et al. 2021). Our data also align with evidence that the posterior parietal cortex, particularly bilateral LIP, becomes increasingly important after a V1 lesion for visually guided saccades within the affected field (Kato et al. 2021) and to sustain visuomotor transformation in manual responses (Celeghin et al. 2017).

#### Feedforward processing without recurrent activity

These pathway-level considerations fit a broader framework in which a V1 lesion degrades the fidelity of sensory evidence used for decision and motor specification without abolishing all action-relevant visual signals (Supèr et al. 2001; Ekstrom et al. 2008; Ekstrom et al. 2009; Goodale 2011; Ioannides et al. 2012; Silvanto 2015). Within this framework, a feedforward mode of processing may survive in the absence of the recurrent activity thought to support visual awareness. Hence, residual V1-independent pathways can still provide signals sufficient for orienting behavior, but those signals are coarse, unreliable, and often insufficient for precise, timely, and consciously accessible localization.

#### Decision-theoretic accounts and the ON–OFF dissociation

Decision-theoretic models provide a complementary interpretation, positing that responses, including saccades, are triggered when accumulated evidence crosses a decision bound (Gold and Shadlen 2007; Kepecs et al. 2008; Martin, Das and Huxlin 2012; Summerfield and de Lange 2014). As outlined above, V1 lesions raise this decision threshold for saccade initiation, making responses less deliberate. In the fixation-ON task, only some scotoma locations elicited saccades above the aggregate catch-trial baseline, suggesting that residual signals at those locations occasionally crossed the threshold for movement initiation. The fixation-OFF task may have effectively lowered this threshold by reducing temporal uncertainty about target onset, expanding the set of locations at which weak residual signals could drive initiation.

### Implications, limitations, and future directions

#### Atypical saccades as a sensitive index of residual blind-field function

A first implication of our findings is that residual functions in the blind can be substantially underestimated when assessment is limited to correct responses alone. Especially in the early postlesion period, informative behavior may be expressed through delayed, incomplete, or initially misdirected saccades. Including such responses in quantitative assessments could provide a more sensitive measure of spared and compensatory visuomotor behavior and a more precise map of heterogeneous response profiles within the scotoma.

This point may be relevant for rehabilitation. Repeated visual discrimination training or feature-based attentional cueing can improve blind-field performance and even Humphrey perimetry (Huxlin et al. 2009; Sahraie et al. 2010; Das et al. 2014; Cavanaugh et al. 2015, 2016, 2019; Cavanaugh and Huxlin 2017). Many current approaches to hemianopia rely on eye movement strategies or relatively coarse outcome measures, in which the blind field is treated as a largely unitary deficit (Ro and Rafal 2006; Chokron et al. 2008; Schuett et al. 2009; Pollock et al. 2019; Sahraie et al. 2020; Zihl et al. 2021). Our results suggest that deficits in saccade initiation, direction specification, timing, and endpoint control may partly dissociate. Patients who produce many Misdirect responses may retain some capacity to initiate orienting into the blind field, but fail to specify the appropriate movement vector accurately. In contrast, patients who produce more Out-of-time-like responses may preserve target-location dependence more faithfully, while failing to satisfy temporal or endpoint constraints. A trajectory-based approach could therefore motivate more individualized training strategies and more sensitive behavioral endpoints, and is consistent with the more general view that residual signals exist before the conditions for their reliable behavioral readout are fully established.

#### Early postlesion testing and the case for longitudinal designs

The study also underscores the importance of early postlesion testing. Because we detected structured residual behavior within only 3 to 4 weeks, these responses are unlikely to depend exclusively on very late reorganization. This translational point is reinforced by human data showing that visual training initiated in the subacute period after occipital stroke can be faster and more spatially generalizable than comparable training initiated in chronic patients, and can preserve visual discriminations that might otherwise deteriorate over time (Saionz et al. 2020). Longitudinal studies will now be needed to determine whether atypical responses gradually evolve into correct responses, remain stable as a distinct residual phenotype, or vary as a function of lesion extent, location, task structure, and practice. Such trajectories could help link acute residual capacity to later plasticity. Recent human studies also suggest that residual visual function and training-induced improvement are related to lesion topology, hMT+ neurochemistry, and white-matter integrity, offering candidate biomarkers that future macaque-human comparisons could test directly (Willis et al. 2023, 2024a, 2024b, 2025, 2025b).

#### Limitations

Several limitations should be acknowledged. First, the study is based on only two animals. Although the overall logic of the results was consistent across them, inter-individual differences in scotoma topography and spatial distribution of residual responses were evident. The present findings should therefore be interpreted as a detailed within-subject characterization of early postlesion visuomotor behavior rather than as an estimate of population-level variability.

Second, the conclusions are based solely on behavior. We therefore cannot directly identify the neural pathways underlying each response class, nor can we fully separate residual visual processing from strategic sampling, reward-probability learning, or other compensatory factors.

Third, the fixation-OFF condition did not include catch trials, because the simultaneous disappearance of the fixation point and appearance of the target constitutes an inherent temporal cue that defines the trial type. Introducing a fixation offset without a target would require rewarding outcomes independently of behavior, thereby undermining the temporal cueing manipulation and altering the reward structure across trial types. This design choice, consistent with the canonical macaque blindsight paradigm (Moore et al. 1995; Yoshida and Isa 2015), means that target-specific false-alarm rates cannot be estimated in the fixation-OFF condition. The absence of an OFF-condition baseline is partially addressed by two controls: the ON–OFF dissociation was absent prelesion at the same locations (Table 2), and ipsilesional targets showed no comparable OFF-related increase postlesion (Fig. S5). Nevertheless, the possibility that fixation offset itself contributed to the increase in blind-field saccades, independently of target presence, cannot be fully excluded and represents a limitation of the present design.

Fourth, testing was conducted under binocular viewing. This precluded the use of the physiological blind spot as an internal control for light scatter, a check available only under monocular conditions (Moore et al. 1995). Although targets were small (0.5°) and the scotoma extended well beyond the parafoveal region where scatter would be most consequential, we cannot fully exclude that some responses to scotoma targets were influenced by light scattered onto spared retinal regions.

#### Future directions

Future work should combine the present trajectory-based phenotyping with longitudinal designs, neural recordings, and causal manipulation of candidate pathways, particularly those involving the SC, pulvinar, LGN, and posterior parietal cortex. The value of the present behavioral taxonomy is to make response-class distinctions measurable at the single-trial level and their neural underpinnings accessible to causal investigation. It will also be important to manipulate reward contingencies and task structure more explicitly to separate residual visual influences from postlesion changes in target-independent saccadic behavior. More broadly, applying the same response taxonomy in macaques and human patients may help establish a common behavioral framework for studying the emergence, stabilization, and possible rehabilitation of blindsight (Tamietto et al. 2026).

## Supplementary Material

Supplementary_Material_bhag138

## Data Availability

Data and code are available upon request.
