# Supplementary material for: Early postlesional visuomotor capacity after V1 damage in the macaque
Source: Cereb Cortex. 2026 Oct 5;36(9):bhag138. doi: 10.1093/cercor/bhag138 (PMC13755703; doi:10.1093/cercor/bhag138)
Supplement: Supplementary file 1 [file supplementary_material_bhag138.docx]

**Early postlesional visuomotor capacity after V1 damage in the macaque**

**Running title:** Early visuomotor capacity after V1 lesion

Alessia Sepe^1,2,3°^, Matteo Panormita^1,2,4°^, Mark A.G. Eldridge^5,6^, David A. Leopold^6,7^, Luca Bonini^3^, Wim Vanduffel^1,2,8,9,‡ *^ and Marco Tamietto^4,10,11‡,*^

^1^Department of Neurosciences, Laboratory of Neuro- and Psychophysiology, KU Leuven Medical School, Leuven, 3000, Belgium

^2^Leuven Brain Institute, KU Leuven; Leuven, 3000, Belgium

^3^Department of Medicine and Surgery, Neuroscience Unit, University of Parma, Parma, Italy

^4^Department of Psychology and Neuroscience Institute of Turin (NIT), University of Torino, Torino, Italy

^5^Biosciences Institute, Newcastle University, Newcastle upon Tyne NE1 7RU, UK

^6^Section on Cognitive Neurophysiology and Imaging, National Institute of Mental Health, Bethesda, MD, USA.

^7^Neurophysiology Imaging Facility, National Institute of Mental Health, National Institute of Neurological Disorders and Stroke, National Eye Institute, Bethesda, MD, USA.

^8^Athinoula A. Martinos Center for Biomedical Imaging, Massachusetts General Hospital, Charlestown, MA, 02129, USA

^9^Department of Radiology, Harvard Medical School, Boston, MA, 02144, USA

^10^Department of Medical and Clinical Psychology, Tilburg University, Tilburg, the Netherlands

^11^Italian Academy for Advanced Studies in America, Columbia University, New York, NY, USA

°These authors contributed equally to the work
‡ Co-senior authors

*Corresponding author:
E-mail: [wim.vanduffel@kuleuven.be](mailto:wim.vanduffel@kuleuven.be); [marco.tamietto@unito.it](mailto:marco.tamietto@unito.it); [m.tamietto@tilburguniversity.edu](mailto:m.tamietto@tilburguniversity.edu)

**Supplementary Information**

**Para-foveal**

**M2**

|  |  | ECC 5 | | | | | | | | ECC 10 | | | | | | | |
| --- | --- | --- | --- | --- | --- | --- | --- | --- | --- | --- | --- | --- | --- | --- | --- | --- | --- |
|  |  | **C** | | **OT** | | **MISD** | | **MISS** | | **C** | | **OT** | | **MISD** | | **MISS** | |
| PA |  | **on** | **off** | **on** | **off** | **on** | **off** | **on** | **off** | **on** | **off** | **on** | **off** | **on** | **off** | **on** | **off** |
| 180 | *mdn* | 9.8 | 30.4 | n.s. | | 9.8 | 32.1 | 80.5 | 37.5 | 84.6 | 100.0 | n.s. | | 12.8 | 0.0 | n.s. | |
|  | *CI 95%* | [5.5, 35.5] | |  |  | [7.9, 38.0] | | [-60.4, -26.1] | | [5.1, 28.2] | |  |  | [-25.6, -2.6] | |  |  |
|  | *t* | 2.64 | |  |  | 2.96 | | -4.87 | | 2.63 | |  |  | -2.36 | |  |  |
|  | *p* | 0.022 | |  |  | 0.004 | | <0.0001 | | <0.0001 | |  |  | 0.006 | |  |  |
| 210 | *mdn* | n.s. | | n.s. | | 7.9 | 49.1 | 92.1 | 45.5 | n.s. | | n.s. | | 17.1 | 40.0 | 80.5 | 54.5 |
|  | *CI 95%* |  |  |  |  | [25.0, 57.4] | | [-61.0, -31.3] | |  |  |  |  | [5.9, 40.5] | | [-43.0, -8.3] | |
|  | *t* |  |  |  |  | 5.17 | | -5.94 | |  |  |  |  | 2.63 | | -2.93 | |
|  | *p* |  |  |  |  | <0.0001 | | <0.0001 | |  |  |  |  | 0.008 | | 0.002 | |
| 240 | *mdn* | n.s. | | n.s. | | 8.8 | 45.5 | 91.2 | 54.5 | n.s. | | n.s. | | 7.9 | 43.6 | 89.5 | 41.8 |
|  | *CI 95%* |  |  |  |  | [19.8, 52.7] | | [-52.7, -19.8] | |  |  |  |  | [18.8, 51.1] | | [-63.0, -29.9] | |
|  | *t* |  |  |  |  | 4.39 | | -4.39 | |  |  |  |  | 4.35 | | -5.58 | |
|  | *p* |  |  |  |  | <0.0001 | | <0.0001 | |  |  |  |  | <0.0001 | | <0.0001 | |

**A**

**M1**

|  |  | ECC 5 | | | | | | | | ECC 10 | | | | | | | |
| --- | --- | --- | --- | --- | --- | --- | --- | --- | --- | --- | --- | --- | --- | --- | --- | --- | --- |
|  |  | **C** | | **OT** | | **MISD** | | **MISS** | | **C** | | **OT** | | **MISD** | | **MISS** | |
| PA |  | **on** | **off** | **on** | **off** | **on** | **off** | **on** | **off** | **on** | **off** | **on** | **off** | **on** | **off** | **on** | **off** |
| 120 | *mdn* | n.s. | | n.s. | | n.s. | | n.s. | | n.s. | | n.s. | | 5.6 | 0.0 | n.s. | |
|  | *CI 95%* |  |  |  |  |  |  |  |  |  |  |  |  | [-13.0, 0.0] | |  |  |
|  | *t* |  |  |  |  |  |  |  |  |  |  |  |  | -1.77 | |  |  |
|  | *p* |  |  |  |  |  |  |  |  |  |  |  |  | 0.049 | |  |  |
| 150 | *mdn* | 0.0 | 14.3 | n.s. | | 6.2 | 54.0 | 93.8 | 31.7 | 89.6 | 98.3 | n.s. | | n.s. | | n.s. | |
|  | *CI 95%* | [6.3, 23.8] | |  |  | [33.4, 60.9] | | [-74.7, -47.8] | | [0.4, 19.1] | |  |  |  |  |  |  |
|  | *t* | 3.24 | |  |  | 6.51 | | -9.08 | | 1.82 | |  |  |  |  |  |  |
|  | *p* | <0.0001 | |  |  | <0.0001 | | <0.0001 | | 0.045 | |  |  |  |  |  |  |
| 180 | *mdn* | 0.0 | 16.7 | n.s. | | n.s. | | 79.6 | 45.5 | 0.0 | 4.8 | 0.0 | 6.5 | 6.8 | 51.6 | 93.2 | 37.1 |
|  | *CI 95%* | [7.6, 25.8] | |  |  |  |  | [-49.9, -16.9] | | [0.0, 9.7] | | [1.6, 12.9] | | [30.6, 59.0] | | [-70.6, -42.2] | |
|  | *t* | 3.59 | |  |  |  |  | -4.05 | | 1.75 | | 2.1 | | 6.23 | | -7.81 | |
|  | *p* | <0.0001 | |  |  |  |  | <0.0001 | | 0.043 | | 0.016 | | <0.0001 | | <0.0001 | |
| 210 | *mdn* | 12.7 | 28.1 | n.s. | | 10.9 | 53.1 | 74.5 | 18.8 | n.s. | | 0.0 | 6.7 | 13.7 | 46.7 | 86.3 | 43.3 |
|  | *CI 95%* | [1.6, 29.2] | |  |  | [27.4, 55.5] | | [-69.8, -41.2] | |  |  | [1.7, 13.3] | | [17.5, 47.8] | | [-58.2, -27.1] | |
|  | *t* | 2.24 | |  |  | 5.88 | | -7.47 | |  |  | 2.05 | | 4.06 | | -5.33 | |
|  | *p* | 0.030 | |  |  | <0.0001 | | <0.0001 | |  |  | 0.019 | | <0.0001 | | <0.0001 | |
| 240 | *mdn* | 16.4 | 36.1 | n.s. | | 12.7 | 41.0 | 70.9 | 19.7 | 3.5 | 15.5 | 0.0 | 6.9 | 8.8 | 44.8 | 87.7 | 32.8 |
|  | *CI 95%* | [5.7, 33.9] | |  |  | [13.3, 42.3] | | [-65.4, -35.2] | | [3.3, 22.4] | | [1.7, 13.8] | | [22.2, 49.9] | | [-68.8, -39.4] | |
|  | *t* | 2.58 | |  |  | 3.62 | | -6.48 | | 2.29 | | 2.01 | | 4.9 | | -7.28 | |
|  | *p* | 0.010 | |  |  | 0.004 | | <0.0001 | | 0.014 | | 0.018 | | <0.0001 | | <0.0001 | |

**B**

**Table S1A-B — Para-foveal target specific statistics for positions within the scotoma (5° & 10° eccentricity)**

Statistics by trial category and polar angle (PA) for para-foveal targets (eccentricities 5° and 10°). For each polar angle (PA; rows show *median*, *CI 95%*, *t-score*, *p-value*) and category—Correct (C), Out-of-Time (OT), Misdirect (MISD), and Miss (MISS)—the table reports the median (mdn) percentage of trials for each category and for the two experimental conditions (ON and OFF), the 95% confidence interval (CI) for the difference between ON and OFF trials, the bootstrapped t-statistic, and the bootstrapped p-value. “n.s.” indicate non-significant effects in that PA×ECC×category cell.

**Mid-Periphery**

**M2**

|  |  | ECC 15 | | | | | | | | | ECC 20 | | | | | | | |
| --- | --- | --- | --- | --- | --- | --- | --- | --- | --- | --- | --- | --- | --- | --- | --- | --- | --- | --- |
|  |  | **C** | | **OT** | | **MISD** | | **MISS** | | **C** | | | **OT** | | **MISD** | | **MISS** | |
| PA |  | **on** | **off** | **on** | **off** | **on** | **off** | **on** | **off** | **on** | | **off** | **on** | **off** | **on** | **off** | **on** | **off** |
| 180 | *mdn* | n.s. | | n.s. | | n.s. | | n.s. | | n.s. | | | n.s. | | n.s. | | 42.5 | 16.4 |
|  | *CI 95%* |  |  |  |  |  |  |  |  |  |  |  |  |  |  |  | [-43.6, -8.4] | |
|  | *t* |  |  |  |  |  |  |  |  |  |  |  |  |  |  |  | -2.96 | |
|  | *p* |  |  |  |  |  |  |  |  |  |  |  |  |  |  |  | 0.006 | |
| 210 | *mdn* | n.s. | | n.s. | | 7.1 | 52.7 | 92.9 | 47.3 | n.s. | | | n.s. | | 10.0 | 57.4 | 87.5 | 40.7 |
|  | *CI 95%* |  |  |  |  | [31.0, 60.0] | | [-60.0, -31.0] | |  |  |  |  |  | [30.6, 62.9] | | [-61.7, -29.4] | |
|  | *t* |  |  |  |  | 5.95 | | -5.95 | |  |  |  |  |  | 5.64 | | -5.43 | |
|  | *p* |  |  |  |  | <0.0001 | | <0.0001 | |  |  |  |  |  | <0.0001 | | <0.0001 | |
| 240 | *mdn* | n.s. | | n.s. | | 7.7 | 44.6 | 92.3 | 55.4 | n.s. | | | n.s. | | 15.8 | 50.0 | 84.2 | 48.2 |
|  | *CI 95%* |  |  |  |  | [20.3, 52.8] | | [-52.8, -20.3] | |  |  |  |  |  | [15.6, 51.0] | | [-52.9, -19.0] | |
|  | *t* |  |  |  |  | 4.54 | | -4.54 | |  |  |  |  |  | 3.76 | | -3.98 | |
|  | *p* |  |  |  |  | <0.0001 | | <0.0001 | |  |  |  |  |  | <0.0001 | | <0.0001 | |

**M1**

|  | |  | ECC 15 | | | | | | | | ECC 20 | | | | | | | |
| --- | --- | --- | --- | --- | --- | --- | --- | --- | --- | --- | --- | --- | --- | --- | --- | --- | --- | --- |
|  |  |  | **C** | | **OT** | | **MISD** | | **MISS** | | **C** | | **OT** | | **MISD** | | **MISS** | |
|  | PA |  | **on** | **off** | **on** | **off** | **on** | **off** | **on** | **off** | **on** | **off** | **on** | **off** | **on** | **off** | **on** | **off** |
|  | 120 | *mdn* | 0.0 | 18.5 | 0.0 | 6.2 | 16.0 | 43.1 | 84.0 | 32.3 | 5.9 | 23.3 | 0.0 | 10.0 | 13.7 | 40.0 | 80.4 | 26.7 |
|  |  | *CI 95%* | [9.2, 27.7] | | [1.5, 12.3] | | [10.5, 41.9] | | [-65.4, -37.5] | | [5.2, 30.8] | | [3.3, 18.3] | | [10.1, 41.6] | | [-66.9, -37.8] | |
|  |  | *t* | 3.93 | | 1.98 | | 3.43 | | -6.98 | | 2.7 | | 2.63 | | 3.28 | | -7.04 | |
|  |  | *p* | <0.0001 | | 0.018 | | <0.0001 | | <0.0001 | | 0.004 | | 0.003 | | 0.002 | | <0.0001 | |
|  | 150 | *mdn* | 60.4 | 83.1 | n.s. | | 13.2 | 0.0 | n.s. | | n.s. | | 42.0 | 71.0 | n.s. | | 24.0 | 0.0 |
|  |  | *CI 95%* | [7.6, 37.6] | |  |  | [-22.6, -5.7] | |  |  |  |  | [11.7, 46.3] | |  |  | [-36.0, -12.0] | |
|  |  | *t* | 2.8 | |  |  | -2.94 | |  |  |  |  | 3.32 | |  |  | -3.87 | |
|  |  | *p* | 0.004 | |  |  | 0.001 | |  |  |  |  | 0.004 | |  |  | <0.0001 | |
|  | 180 | *mdn* | 0.0 | 4.9 | n.s. | | 18.6 | 47.5 | 81.4 | 44.3 | n.s. | | 0.0 | 8.5 | 12.5 | 44.1 | 87.5 | 47.5 |
|  |  | *CI 95%* | [0.0, 11.5] | |  |  | [12.4, 44.4] | | [-52.8, -20.1] | |  |  | [1.7, 15.3] | | [16.2, 48.0] | | [-57.0, -22.7] | |
|  |  | *t* | 1.76 | |  |  | 3.45 | | -4.41 | |  |  | 2.41 | | 3.89 | | -4.9 | |
|  |  | *p* | 0.046 | |  |  | <0.0001 | | <0.0001 | |  |  | 0.002 | | <0.0001 | | <0.0001 | |
|  | 210 | *mdn* | 0.0 | 12.7 | 3.8 | 15.9 | 15.4 | 36.5 | 80.8 | 34.9 | n.s. | | 0.0 | 4.7 | 8.7 | 51.6 | 91.3 | 43.8 |
|  |  | *CI 95%* | [6.3, 22.2] | | [1.2, 21.9] | | [6.4, 35.7] | | [-61.5, -30.2] | |  |  | [0.0, 10.9] | | [28.2, 57.2] | | [-62.2, -32.3] | |
|  |  | *t* | 3.04 | | 2.27 | | 2.73 | | -5.68 | |  |  | 1.8 | | 5.69 | | -6.26 | |
|  |  | *p* | <0.0001 | | 0.032 | | 0.004 | | <0.0001 | |  |  | 0.046 | | <0.0001 | | <0.0001 | |
|  | 240 | *mdn* | 0.0 | 10.0 | n.s. | | 8.9 | 51.7 | 91.1 | 38.3 | n.s. | | 0.0 | 5.0 | 6.2 | 51.7 | 93.8 | 41.7 |
|  |  | *CI 95%* | [3.3, 18.3] | |  |  | [28.9, 58.0] | | [-66.6, -38.8] | |  |  | [0.0, 11.7] | | [30.0, 60.0] | | [-66.7, -37.5] | |
|  |  | *t* | 2.51 | |  |  | 5.72 | | -7.13 | |  |  | 1.79 | | 5.99 | | -7.0 | |
|  |  | *p* | 0.002 | |  |  | <0.0001 | | <0.0001 | |  |  | 0.045 | | <0.0001 | | <0.0001 | |

**Table S2A-B — Mid-peripheral target specific statistics for positions within the scotoma (15° & 20° eccentricity)**

Statistics by category and polar angle (PA) for para-foveal targets (eccentricities 15° and 20°). For each PA (rows grouped as *mdn*, *CI 95%*, *t*, *p*) and category—Correct (C), Out-of-Time (OT), Misdirect (MISD), and Miss (MISS)—the table reports the median percentage of trials in the two experimental conditions (ON and OFF), the 95% confidence interval (CI) for the ON-OFF difference, the boostrapped t-statistic, and the bootstrapped p-value. “n.s.” indicate non-significant effects in that PA×ECC×category cell.

**Far Periphery**

**M2**

|  |  | ECC 25 | | | | | | | | ECC 30 | | | | | | | | ECC 35 | | | | | | | |
| --- | --- | --- | --- | --- | --- | --- | --- | --- | --- | --- | --- | --- | --- | --- | --- | --- | --- | --- | --- | --- | --- | --- | --- | --- | --- |
|  |  | **C** | | **OT** | | **MISD** | | **MISS** | | **C** | | **OT** | | **MISD** | | **MISS** | | **C** | | **OT** | | **MISD** | | **MISS** | |
| PA |  | **on** | **off** | **on** | **off** | **on** | **off** | **on** | **off** | **on** | **off** | **on** | **off** | **on** | **off** | **on** | **off** | **on** | **off** | **on** | **off** | **on** | **off** | **on** | **off** |
| 180 | *mdn* | n.s. | | n.s. | | 16.7 | 54.5 | 81.0 | 38.2 | 0.0 | 5.6 | 0.0 | 9.3 | 5.0 | 38.9 | 95.0 | 46.3 | 7.9 | 23.6 | n.s. | | n.s. | | 39.5 | 10.9 |
|  | *CI 95%* |  |  |  |  | [20.1, 54.1] | | [-59.0, -23.6] | | [0.0, 11.1] | | [1.9, 18.5] | | [18.4, 48.1] | | [-64.2, -33.9] | | [0.6, 30.1] | |  |  |  |  | [-45.4, -12.0] | |
|  | *t* |  |  |  |  | 4.23 | | -4.7 | | 1.81 | | 2.33 | | 4.47 | | -6.31 | | 2.08 | |  |  |  |  | -3.19 | |
|  | *p* |  |  |  |  | <0.0001 | | <0.0001 | | 0.045 | | 0.009 | | <0.0001 | | <0.0001 | | 0.044 | |  |  |  |  | <0.0001 | |
| 210 | *mdn* | n.s. | | n.s. | | 14.6 | 43.6 | 82.9 | 52.7 | n.s. | | n.s. | | 2.6 | 30.4 | 94.9 | 69.6 | n.s. | | 0.0 | 5.5 | 6.1 | 40.0 | 93.9 | 54.5 |
|  | *CI 95%* |  |  |  |  | [10.8, 45.4] | | [-47.2, -12.5] | |  |  |  |  | [14.5, 40.3] | | [-38.0, -11.4] | |  |  | [0.0, 12.7] | | [19.4, 47.9] | | [-53.9, -24.8] | |
|  | *t* |  |  |  |  | 3.31 | | -3.38 | |  |  |  |  | 4.35 | | -3.69 | |  |  | 1.72 | | 4.5 | | -5.25 | |
|  | *p* |  |  |  |  | 0.002 | | <0.0001 | |  |  |  |  | <0.0001 | | 0.004 | |  |  | 0.043 | | <0.0001 | | <0.0001 | |
| 240 | *mdn* | n.s. | | n.s. | | 7.1 | 57.4 | 92.9 | 42.6 | n.s. | | n.s. | | 4.9 | 43.6 | 92.7 | 54.5 | n.s. | | 0.0 | 5.5 | 9.3 | 40.0 | 90.7 | 54.5 |
|  | *CI 95%* |  |  |  |  | [35.4, 64.3] | | [-64.3, -35.4] | |  |  |  |  | [23.6, 53.3] | | [-53.3, -22.4] | |  |  | [0.0, 12.7] | | [14.6, 47.6] | | [-52.5, -20.1] | |
|  | *t* |  |  |  |  | 6.74 | | -6.74 | |  |  |  |  | 5.17 | | -4.79 | |  |  | 1.78 | | 3.66 | | -4.3 | |
|  | *p* |  |  |  |  | <0.0001 | | <0.0001 | |  |  |  |  | <0.0001 | | <0.0001 | |  |  | 0.041 | | <0.0001 | | <0.0001 | |

**M1**

|  |  | ECC 25 | | | | | | | | ECC 30 | | | | | | | | ECC 35 | | | | | | | |
| --- | --- | --- | --- | --- | --- | --- | --- | --- | --- | --- | --- | --- | --- | --- | --- | --- | --- | --- | --- | --- | --- | --- | --- | --- | --- |
|  |  | **C** | | **OT** | | **MISD** | | **MISS** | | **C** | | **OT** | | **MISD** | | **MISS** | | **C** | | **OT** | | **MISD** | | **MISS** | |
| PA |  | **on** | **off** | **on** | **off** | **on** | **off** | **on** | **off** | **on** | **off** | **on** | **off** | **on** | **off** | **on** | **off** | **on** | **off** | **on** | **off** | **on** | **off** | **on** | **off** |
| 120 | *mdn* | 26.0 | 46.9 | n.s. | | n.s. | | 18.0 | 0.0 | n.s. | | n.s. | | 6.1 | 0.0 | n.s. | | n.s. | | n.s. | | n.s. | | n.s. | |
|  | *CI 95%* | [2.2, 37.8] | |  |  |  |  | [-28.1, -8.0] | |  |  |  |  | [-14.3, 0.0] | |  |  |  |  |  |  |  |  |  |  |
|  | *t* | 2.28 | |  |  |  |  | -3.38 | |  |  |  |  | -1.78 | |  |  |  |  |  |  |  |  |  |  |
|  | *p* | 0.028 | |  |  |  |  | <0.0001 | |  |  |  |  | 0.042 | |  |  |  |  |  |  |  |  |  |  |
| 150 | *mdn* | n.s. | | n.s. | | n.s. | | n.s. | | n.s. | | n.s. | | n.s. | | n.s. | | 3.8 | 16.1 | 65.4 | 82.3 | n.s. | | 25.0 | 0.0 |
|  | *CI 95%* |  |  |  |  |  |  |  |  |  |  |  |  |  |  |  |  | [1.7, 22.0] | | [0.9, 33.3] | |  |  | [-36.5, -13.5] | |
|  | *t* |  |  |  |  |  |  |  |  |  |  |  |  |  |  |  |  | 2.41 | | 2.02 | |  |  | -4.07 | |
|  | *p* |  |  |  |  |  |  |  |  |  |  |  |  |  |  |  |  | 0.014 | | 0.042 | |  |  | <0.0001 | |
| 180 | *mdn* | n.s. | | 14.3 | 50.0 | 8.2 | 28.3 | 77.6 | 21.7 | n.s. | | 14.8 | 50.0 | n.s. | | 64.8 | 16.1 | 0.0 | 6.7 | 11.8 | 73.3 | n.s. | | 86.3 | 11.7 |
|  | *CI 95%* |  |  | [19.6, 52.2] | | [5.7, 34.3] | | [-70.3, -40.5] | |  |  | [20.2, 50.9] | |  |  | [-63.0, -32.7] | | [1.7, 13.3] | | [46.5, 75.8] | |  |  | [-86.1, -61.0] | |
|  | *t* |  |  | 4.36 | | 2.77 | | -6.95 | |  |  | 4.46 | |  |  | -6.19 | | 2.09 | | 8.44 | |  |  | -11.84 | |
|  | *p* |  |  | <0.0001 | | 0.008 | | <0.0001 | |  |  | <0.0001 | |  |  | <0.0001 | | 0.012 | | <0.0001 | |  |  | <0.0001 | |
| 210 | *mdn* | n.s. | | n.s. | | 10.6 | 52.3 | 89.4 | 44.6 | n.s. | | n.s. | | 12.5 | 49.2 | 87.5 | 47.5 | n.s. | | n.s. | | 2.2 | 56.7 | 97.8 | 43.3 |
|  | *CI 95%* |  |  |  |  | [26.6, 56.1] | | [-59.8, -30.7] | |  |  |  |  | [21.8, 51.4] | | [-55.2, -25.5] | |  |  |  |  | [42.2, 67.8] | | [-67.8, -42.2] | |
|  | *t* |  |  |  |  | 5.38 | | -5.81 | |  |  |  |  | 4.83 | | -5.31 | |  |  |  |  | 8.03 | | -8.03 | |
|  | *p* |  |  |  |  | <0.0001 | | <0.0001 | |  |  |  |  | <0.0001 | | <0.0001 | |  |  |  |  | <0.0001 | | <0.0001 | |
| 240 | *mdn* | n.s. | | 0.0 | 5.1 | 12.2 | 62.7 | 87.8 | 30.5 | n.s. | | 0.0 | 6.6 | 2.1 | 55.7 | 97.9 | 37.7 | n.s. | | 0.0 | 6.6 | 8.7 | 50.8 | 91.3 | 42.6 |
|  | *CI 95%* |  |  | [0.0, 10.2] | | [35.2, 65.4] | | [-70.8, -42.3] | |  |  | [1.6, 13.1] | | [40.0, 66.7] | | [-72.8, -47.1] | |  |  | [1.6, 13.1] | | [26.3, 57.4] | | [-64.0, -32.9] | |
|  | *t* |  |  | 1.77 | | 6.59 | | -7.59 | |  |  | 2.05 | | 7.86 | | -9.15 | |  |  | 2.09 | | 5.52 | | -6.45 | |
|  | *p* |  |  | 0.045 | | <0.0001 | | <0.0001 | |  |  | 0.015 | | <0.0001 | | <0.0001 | |  |  | 0.022 | | <0.0001 | | <0.0001 | |

**Table S3A-B — Far periphery target specific statistics for positions within the scotoma (25°, 30° & 35° eccentricity)**

Statistics by category and polar angle (PA) for para-foveal targets (eccentricities 5° and 10°). For each PA (rows grouped as *mdn*, *CI 95%*, *t*, *p*) and category—Correct (C), Out-of-Time (OT), Misdirect (MISD), and Miss (MISS)—the table reports the median percentage of trials in the two experimental conditions (ON and OFF), the 95% confidence interval (CI) for the ON-OFF difference, the boostrapped t-statistic, and the bootstrapped p-value. “n.s.” indicate non-significant effects in that PA×ECC×category cell.

**M1**

**M2**

**Figure** **S1 A- B**

(**S1A**: M1, **S1B**: M2). OFF–ON percentage differences by target for each response category in the post-lesion dataset. Each panel shows, across targets (x-axis; Eccentricity, Angle), the change in proportion (OFF–ON, y-axis) for one category: Correct (**a**), Out-of-time (**b**), Misdirect (**c**), and Miss (**d**). Bars in red indicate targets with a statistically significant difference (p<0.05); gray bars show non-significant differences. The horizontal line at 0 marks no change.

**Figure S2**

Box Plots showing the distribution of response percentages in pre-lesion dataset targets for yes/no ON (blue) vs. forced-choice OFF (red) conditions in each monkey, separately for Correct, Out-of-time, Misdirect, and Miss saccades. Black diamond marks the mean and the horizontal red line the median. Brackets report ON–OFF significance for each category targets aggregate (n.s., * = p<0.05, ** = p<0.01, *** p<0.001). The forced-choice OFF task didn’t produce any significative increases in none of the categories assessed.

**Figure S3**

**Catch trial performance before and the lesion.** Percentage of catch trials with correct fixation and fixation breaks for M2 and M1 respectively during pre-lesion and post-lesion sessions. Statistical comparison between pre- and post-lesion proportions of correct responses was performed using an exact two-sided binomial test, with the null hypothesis that the post-lesion proportion equals the pre-lesion proportion. significance levels:  **p < 0.001=***.**

**Figure S4.**

**Spatial distribution of exploratory responses to targets during fixation-ON task, above the error rate during post-lesional catch trials.** Polar scatter plot showing target locations within the scotoma where the proportion of “exploratory” responses significantly exceeded catch-trials error rate (M1=6.75%; M2=7.5%) after Benjamini–Hochberg FDR correction (α = 0.05). Each point represents a target location that was statistically significant in a one-sided exact binomial test comparing "Miss" responses compared to the combination of correct, out-of-time and misdirect trials (unrewarded Misdirect trials excluded). FDR correction was applied for fixation-ON trials only for targets within the scotoma. Target positions are shown in polar coordinates with 0° as the right horizontal meridian, angles increasing counterclockwise, and radial distance representing eccentricity in degrees of visual angle (see labels adjacent to each significant target).

**Figure S5**
Box Plots showing the distribution of response percentages in post-lesion dataset for ipsilesional targets (PA 0-90; 270-330) for yes/no ON (blue) vs. forced-choice OFF (red) conditions in each monkey, separately for Correct, Out-of-time, Misdirect, and Miss saccades. Black diamond marks the mean and the horizontal red line the median. Brackets report ON–OFF significance for each category targets aggregate (n.s., * = p<0.05, ** = p<0.01, *** p<0.001). The forced-choice OFF task didn’t produce any significative increases in none of the categories assessed.

**A**

| **Eccentricity** | **Polar angle** | **Expl. (%)** | **Miss (%)** | **CI 95% lower** | **CI 95% upper** | **Cohen's h** | **BH-FDR p-value** |
| --- | --- | --- | --- | --- | --- | --- | --- |
| 5 | 150° | 6.25 | 93.75 | 0.0215 | 0.1684 | -0.0203 | 0.671 |
| 5 | 180° | 20.41 | 79.59 | 0.1148 | 0.3364 | 0.4118 | **0.0027** |
| 5 | 210° | 25.45 | 74.55 | 0.1581 | 0.3830 | 0.5320 | **<0.001** |
| 5 | 240° | 29.09 | 70.91 | 0.1877 | 0.4214 | 0.6137 | **<0.001** |
| 10 | 150° | 97.92 | 2.08 | 0.8910 | 0.9963 | 23.263 | **<0.001** |
| 10 | 180° | 6.82 | 93.18 | 0.0235 | 0.1823 | 0.0027 | 0.642 |
| 10 | 210° | 13.73 | 86.27 | 0.0681 | 0.2572 | 0.2334 | 0.081 |
| 10 | 240° | 12.28 | 87.72 | 0.0608 | 0.2325 | 0.1904 | 0.126 |
| 15 | 120° | 16.00 | 84.00 | 0.0834 | 0.2851 | 0.2974 | **0.029** |
| 15 | 180° | 18.60 | 81.40 | 0.0974 | 0.3262 | 0.3663 | **0.012** |
| 15 | 210° | 19.23 | 80.77 | 0.1080 | 0.3190 | 0.3823 | **0.0039** |
| 15 | 240° | 8.93 | 91.07 | 0.0387 | 0.1926 | 0.0812 | 0.396 |
| 20 | 120° | 19.61 | 80.39 | 0.1102 | 0.3246 | 0.3918 | **0.0035** |
| 20 | 150° | 76.00 | 24.00 | 0.6259 | 0.8570 | 15.920 | **<0.001** |
| 20 | 180° | 12.50 | 87.50 | 0.0586 | 0.2470 | 0.1971 | 0.137 |
| 20 | 210° | 8.70 | 91.30 | 0.0343 | 0.2032 | 0.0730 | 0.431 |
| 20 | 240° | 6.25 | 93.75 | 0.0215 | 0.1684 | -0.0203 | 0.671 |
| 25 | 120° | 82.00 | 18.00 | 0.6920 | 0.9023 | 17.396 | **<0.001** |
| 25 | 150° | 96.00 | 4.00 | 0.8654 | 0.9890 | 22.132 | **<0.001** |
| 25 | 180° | 22.45 | 77.55 | 0.1302 | 0.3588 | 0.4616 | **<0.001** |
| 25 | 210° | 10.64 | 89.36 | 0.0463 | 0.2259 | 0.1388 | 0.262 |
| 25 | 240° | 12.24 | 87.76 | 0.0573 | 0.2424 | 0.1893 | 0.143 |
| 25 | 90° | 90.20 | 9.80 | 0.7902 | 0.9574 | 19.790 | **<0.001** |
| 30 | 150° | 96.30 | 3.70 | 0.8746 | 0.9898 | 22.286 | **<0.001** |
| 30 | 180° | 35.19 | 64.81 | 0.2382 | 0.4852 | 0.7443 | **<0.001** |
| 30 | 210° | 12.50 | 87.50 | 0.0586 | 0.2470 | 0.1971 | 0.137 |
| 30 | 240° | 2.13 | 97.87 | 0.0038 | 0.1111 | -0.2329 | 0.963 |
| 35 | 120° | 95.74 | 4.26 | 0.8575 | 0.9883 | 22.004 | **<0.001** |
| 35 | 150° | 75.00 | 25.00 | 0.6179 | 0.8477 | 15.687 | **<0.001** |
| 35 | 180° | 13.73 | 86.27 | 0.0681 | 0.2572 | 0.2334 | 0.081 |
| 35 | 210° | 2.22 | 97.78 | 0.0039 | 0.1157 | -0.2264 | 0.963 |
| 35 | 240° | 8.70 | 91.30 | 0.0343 | 0.2032 | 0.0730 | 0.431 |

**B**

| **Eccentricity** | **Polar angle** | **Expl. (%)** | **Miss (%)** | **CI 95% lower** | **CI 95% upper** | **Cohen's h** | **BH-FDR p-value** |
| --- | --- | --- | --- | --- | --- | --- | --- |
| 5 | 180° | 19.51 | 80.49 | 0.1023 | 0.3401 | 0.3602 | **0.035** |
| 5 | 210° | 7.89 | 92.11 | 0.0272 | 0.2080 | 0.0148 | 0.722 |
| 5 | 240° | 8.82 | 91.18 | 0.0305 | 0.2296 | 0.0484 | 0.722 |
| 10 | 210° | 19.51 | 80.49 | 0.1023 | 0.3401 | 0.3602 | **0.035** |
| 10 | 240° | 10.53 | 89.47 | 0.0417 | 0.2413 | 0.1060 | 0.607 |
| 15 | 180° | 87.80 | 12.20 | 0.7446 | 0.9468 | 18.733 | **<0.001** |
| 15 | 210° | 7.14 | 92.86 | 0.0246 | 0.1901 | -0.0137 | 0.722 |
| 15 | 240° | 7.69 | 92.31 | 0.0265 | 0.2032 | 0.0073 | 0.722 |
| 20 | 180° | 57.50 | 42.50 | 0.4220 | 0.7149 | 11.666 | **<0.001** |
| 20 | 210° | 12.50 | 87.50 | 0.0546 | 0.2611 | 0.1679 | 0.374 |
| 20 | 240° | 15.79 | 84.21 | 0.0744 | 0.3042 | 0.2625 | 0.145 |
| 25 | 180° | 19.05 | 80.95 | 0.0998 | 0.3330 | 0.3485 | **0.035** |
| 25 | 210° | 17.07 | 82.93 | 0.0853 | 0.3126 | 0.2971 | 0.082 |
| 25 | 240° | 7.14 | 92.86 | 0.0246 | 0.1901 | -0.0137 | 0.722 |
| 30 | 180° | 5.00 | 95.00 | 0.0138 | 0.1650 | -0.1038 | 0.812 |
| 30 | 210° | 5.13 | 94.87 | 0.0142 | 0.1689 | -0.0979 | 0.812 |
| 30 | 240° | 7.32 | 92.68 | 0.0252 | 0.1943 | -0.0070 | 0.722 |
| 35 | 180° | 60.53 | 39.47 | 0.4472 | 0.7440 | 12.281 | **<0.001** |
| 35 | 210° | 6.06 | 93.94 | 0.0168 | 0.1961 | -0.0573 | 0.795 |
| 35 | 240° | 9.30 | 90.70 | 0.0368 | 0.2160 | 0.0651 | 0.709 |

**Tables S4A-B (M1 and M2 respectively).** For each target location within the scotoma, Expl. (%) represents the percentage of trials with exploratory responses (Correct, Out-of-time and Misdirect), while Miss (%) represents the percentage of trials with target misses. CI 95% lower and CI 95% upper denote the Wilson score 95% confidence intervals for the exploratory proportion. Cohen's h quantifies the effect size comparing the observed exploratory proportion against the null hypothesis threshold of 6.75% for M1 and 7.5% for M2. BH-FDR p-value indicates the Benjamini-Hochberg false discovery rate-corrected p-value from one-sided exact binomial tests evaluating whether the exploratory proportion significantly exceeds the two thresholds.
